# Arene C−H Activation at Aluminium(I): *meta* Selectivity Driven by the Electronics of S_N_Ar Chemistry

**DOI:** 10.1002/anie.202008557

**Published:** 2020-09-02

**Authors:** Jamie Hicks, Petra Vasko, Andreas Heilmann, Jose M. Goicoechea, Simon Aldridge

**Affiliations:** ^1^ Inorganic Chemistry Laboratory Department of Chemistry University of Oxford South Parks Road Oxford OX1 3QR UK; ^2^ Research School of Chemistry Australian National University, Building 137 Sullivan's Creek Road Acton ACT 2601 Australia; ^3^ Department of Chemistry Nanoscience Center University of Jyväskylä P. O. Box 35 40014 Jyväskylä Finland

**Keywords:** aluminum, aluminyl nucleophiles, arenes, C−H activation, S_N_Ar mechanism

## Abstract

The reactivity of the electron‐rich anionic Al^I^ aluminyl compound K_2_[(NON)Al]_2_ (NON=4,5‐bis(2,6‐diisopropylanilido)‐2,7‐di‐*tert*‐butyl‐9,9‐dimethylxanthene) towards mono‐ and disubstituted arenes is reported. C−H activation chemistry with *n*‐butylbenzene gives exclusively the product of activation at the arene *meta* position. Mechanistically, this transformation proceeds in a single step via a concerted Meisenheimer‐type transition state. Selectivity is therefore based on similar electronic factors to classical S_N_Ar chemistry, which implies the destabilisation of transition states featuring electron‐donating groups in either *ortho* or *para* positions. In the cases of toluene and the three isomers of xylene, benzylic C−H activation is also possible, with the product(s) formed reflecting the feasibility (or otherwise) of competing arene C−H activation at a site which is neither *ortho* nor *para* to a methyl substituent.

## Graphical abstract

Exclusive *meta* selectivity was observed in C−H activation reactions of an electron‐rich Al^I^ aluminyl compound with arenes (see scheme). The selectivity is based on similar electronic factors to those that determine the course of classical S_N_Ar reactions, which implies the destabilisation of transition states featuring electron‐donating groups in *ortho* or *para* positions.

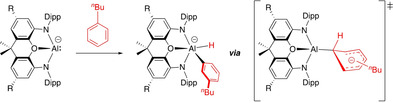

The functionalisation of unactivated C−H bonds has long been regarded as one of the “Holy Grails” of organometallic chemistry and remains the subject of significant research effort today.^[1]^ The idea of exploiting this near ubiquitous “functional group” to build molecular complexity represents an attractive synthetic paradigm, but one that brings with it inherent challenges stemming from issues of selectivity.^[2]^ Oxidative addition at a transition metal centre represents a widely investigated mechanism by which initial activation of a C−H bond can be effected.^[3]^ A primary driver of selectivity in many cases are steric factors, reflecting a relatively late transition state, that is, the close approach to the metal centre required to effect significant transfer of electron density to the C−H σ* orbital.^[4]^

While Green's seminal study reporting the oxidative addition of the C−H bond in benzene at tungsten was reported in 1970,^[5]^ the accomplishment of an analogous transformation at a main group metal centre was reported much more recently.^[6]^ Main group compounds which can activate benzene by deprotonation are well known,^[7]^ (and *meta* selectivity has been achieved for some arene substrates by employing templated systems),^[8]^ but the formal oxidative addition of C−H bonds at a single main group metal centre was for many years limited to more activated hydrocarbon substrates.^[9]^

In 2018 we reported the synthesis of the anionic Al^I^ aluminyl compound **1**,^[6]^ which is sufficiently electron‐rich to act as a metal‐centred nucleophile towards C−X and M−X bonds,^[10]^ and, in addition, reacts with benzene at 60 °C via formal C−H oxidative addition to give the corresponding Al^III^ phenyl hydride (**2**, Scheme 1).^[6]^ Subsequently, a number of other aluminyl compounds have been reported,^[11]^ including others which will effect a similar transformation with benzene.[^11b^, ^11e^, ^12^] With this in mind we were keen to explore the scope and selectivity for C−H activation chemistry of **1** with substituted arenes. These studies are reported in the current manuscript.

**Scheme 1:**
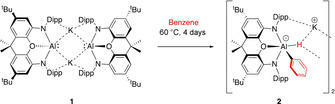
Formal oxidative addition of the C−H bond in benzene at the Al^I^ centres in potassium aluminyl complex **1**.^[6]^ Dipp=2,6‐diisopropylphenyl.

Initial experiments focused on the reactivity of the potassium aluminyl dimer **1** with the monosubstituted arenes toluene, methoxybenzene (anisole) and bromobenzene. The reaction with toluene requires similar conditions to benzene: heating **1** in neat arene solvent at 80 °C for 2 days leads to complete consumption of the aluminyl starting material. The products, both resulting from C−H bond activation, have been unambiguously characterised using spectroscopic, analytical and crystallographic methods (Scheme 2).

**Scheme 2:**
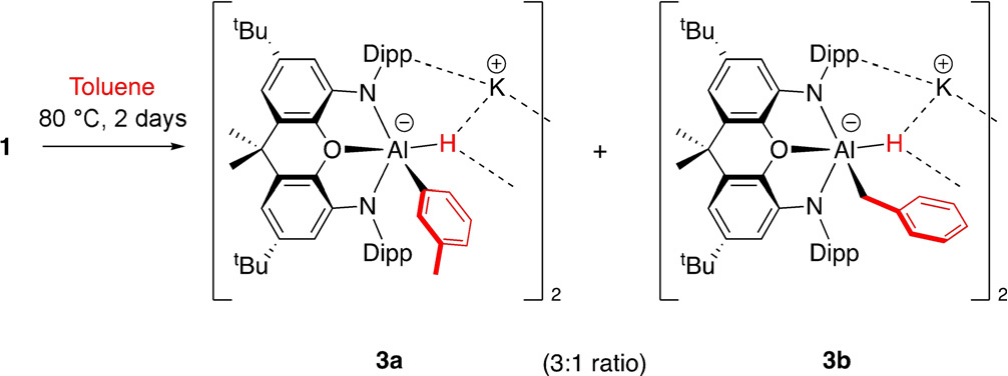
Reaction of potassium aluminyl dimer **1** with toluene proceeding through formal C−H oxidative addition at Al^I^.

Two species can be identified in a 3:1 ratio both in situ (e.g. by ^1^H NMR monitoring), and in the final recrystallised product (both crystallographically and spectroscopically). These are shown to result from *meta*‐aryl (**3 a**) and benzylic C−H bond activation (**3 b**); no hint of the corresponding *ortho*‐ or *para*‐C−H activation products is obtained either from in situ measurements, or in the isolated product mixture. **3 a**/**3 b** cocrystallise, with the dimeric structural motif revealed crystallographically being similar to that found for the benzene activation product **2** (Figure 1). Within the symmetrical dimer, both anionic sites are occupied by the aryl activation products [(NON)AlH(C_6_H_4_Me‐3)]^−^ (NON=4,5‐bis(2,6‐diisopropylanilido)‐2,7‐di‐*tert*‐butyl‐9,9‐dimethylxanthene) and the benzyl isomer [(NON)AlH(CH_2_Ph)]^−^ in a 3:1 ratio, giving an overall composition reflecting the ratio of **3 a**:**3 b** obtained from NMR measurements (also 3:1). Structurally, the aluminium centres in **3 a**/**3 b** and **2** are very similar,^[6]^ featuring a five‐coordinate metal geometry which lies between square pyramidal and trigonal bipyramidal, with due allowance made for the uncertainty in the H‐atom locations (e.g. *τ*=0.59 for **3 a**). Viewed from the square pyramidal limit, the N, O and H donors constitute an approximate basal plane, to which the Al−C bond adopts a roughly perpendicular alignment.

**Figure 1:**
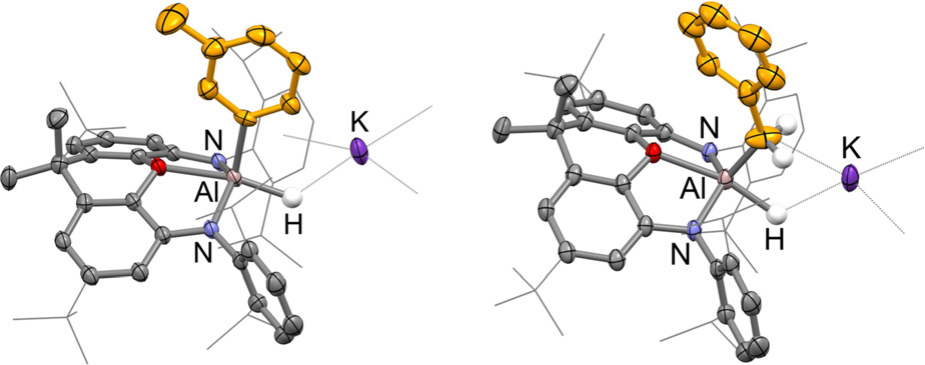
Molecular structures of **3 a** (left) and **3 b** (right) within the dimeric unit as determined by X‐ray crystallography. Thermal ellipsoids set at the 50 % probability level. Solvate molecules and most H atoms omitted, and selected groups shown in wireframe format for clarity. Selected bond lengths [Å] and angles [°] for **3 a**: Al–O 2.146(2), Al–N 1.937(2), 1.938(2), Al–C 2.053(5), Al–H 1.77(5); N‐Al‐N 129.2(1); for **3 b**: Al–C 1.82(2).^[15]^

The corresponding reactions with anisole and bromobenzene were investigated with a view to probing electronic effects on the reactivity of **1** with arenes. Under otherwise identical conditions, the reaction with anisole proceeds much more rapidly (completion in 30 min), but can be shown by spectroscopic and crystallographic methods to proceed not via C−H activation, but via the cleavage of a C−O bond, to generate aluminium‐bound methyl and phenoxy substituents (i.e. **4**, Scheme 3). While this chemistry formally constitutes oxidative addition of the C−O bond at Al^I^,^[13]^ we propose, based on the reactivity of **1** towards MeI and MeOTf,^[6]^ that it proceeds via *S*_N_2 nucleophilic attack on the methyl group of anisole with ejection of PhO^−^. Subsequent retention of the phenoxide group within the coordination sphere of aluminium (in contrast to the precipitation of KI/KOTf in the reactions with MeI/MeOTf) presumably reflects the differing coordination capabilities of PhO^−^ compared to I^−^ and OTf^−^ at Al^III^. The reaction with bromobenzene (see ESI) also proceeds very rapidly, in this case by C−Br activation presumably through a S_N_Ar mechanism akin to that seen with benzene (see below).^[14]^

**Scheme 3:**
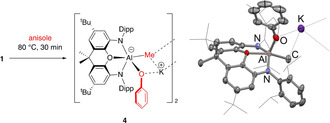
Left: Reaction of **1** with methoxybenzene (anisole) proceeding through formal C−O oxidative addition at Al^I^. Right: Molecular structure of **4** within the dimeric unit as determined by X‐ray crystallography. Thermal ellipsoids set at the 50 % probability level. H atoms omitted and selected groups shown in wireframe format for clarity. Selected bond lengths [Å] and angles [°]: Al–O 2.122(1), Al–N 1.936(2), 1.939(1), Al–C 2.009(3), Al–O_Ph_ 1.799(2); N‐Al‐N 134.6(1).^[15]^

Given the regioselectivity observed in the reaction of **1** with toluene, we were interested in its implications in the corresponding chemistry with disubstituted arenes. Accordingly, the reactions of **1** with *ortho*‐, *meta*‐ and *para*‐xylenes have been investigated (Scheme 4) under similar reaction conditions. The results of these experiments are consistent with those obtained with toluene, that is, among arene C−H bonds, only the *meta* positions are activated. As such, with substrates such as the *ortho* and *para* isomers of xylene (in which *all* arene C−H groups are either *ortho* or *para* to one of the methyl substituents) no detectable aryl–aluminium products are formed. In the case of *ortho*‐xylene the exclusive product, K_2_[(NON)AlH{CH_2_(C_6_H_4_Me‐2)}]_2_ (**5**), results from activation at the benzylic C−H position (Scheme 4 and Figure 2). Similar considerations apply to the reaction with *para*‐xylene, although in this case the initially formed [(NON)AlH{CH_2_(C_6_H_4_Me‐4)]^−^ moiety readily eliminates *para*‐xylylene (which oligomerises) and gives the known dihydroaluminate K_2_[(NON)AlH_2_]_2_ (**7**)^[6]^ as the aluminium‐containing product (Scheme 4 and ESI). In the case of *meta*‐xylene, which (uniquely) *does* feature an arene C−H site that is neither *ortho* or *para* to a methyl group, activation at the mutually *meta* (5‐) position competes with benzylic activation, leading to the formation of a ca. 2:3 mixture of aryl and benzyl isomers **6 a** and **6 b** (Scheme 4 and Figure 2).

**Figure 2:**
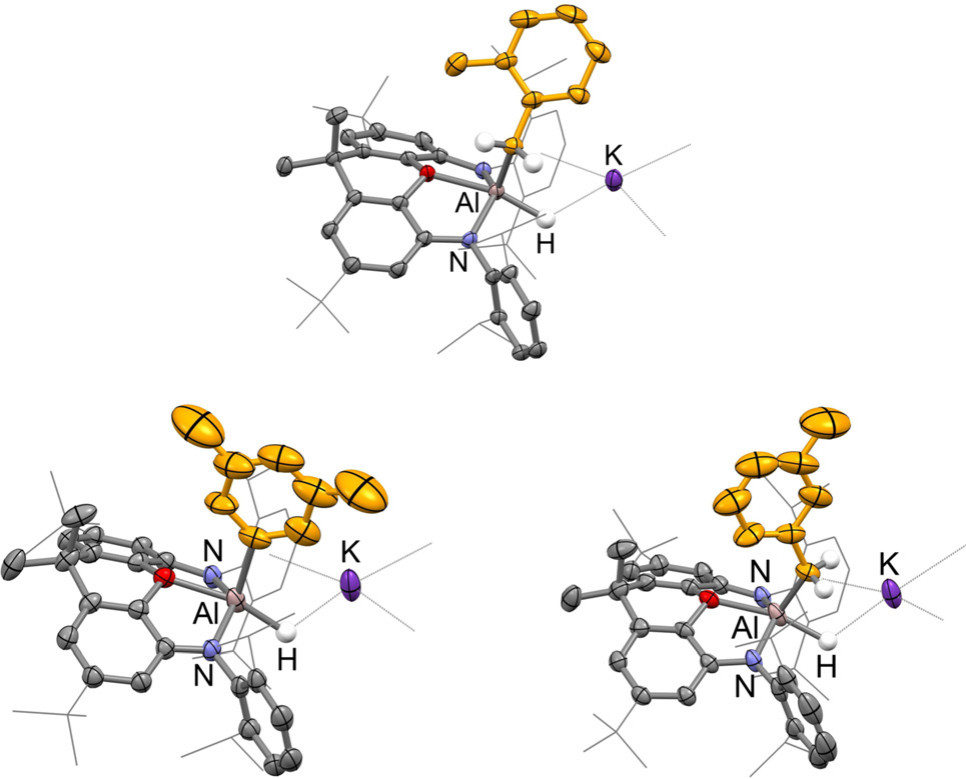
Molecular structures of **5** (top), **6 a** (bottom left), and **6 b** (bottom right) within the dimeric unit as determined by X‐ray crystallography. Thermal ellipsoids set at the 50 % probability level. Solvate molecules and most H atoms omitted, and selected groups shown in wireframe format for clarity. Selected bond lengths [Å] and angles [°] for **5**: Al–O 2.127(2), Al–N 1.946(2), 1.958(1), Al–C 2.081(3), Al–H 1.73(5); N‐Al‐N 130.6(1); for **6 b**: Al–O 2.152(2), Al–N 1.930(3), 1.935(3), Al–C 1.956(6), Al–H 1.81(6); N‐Al‐N 130.2(1); for **6 a**: Al–C 2.25(1).^[15]^

**Scheme 4:**
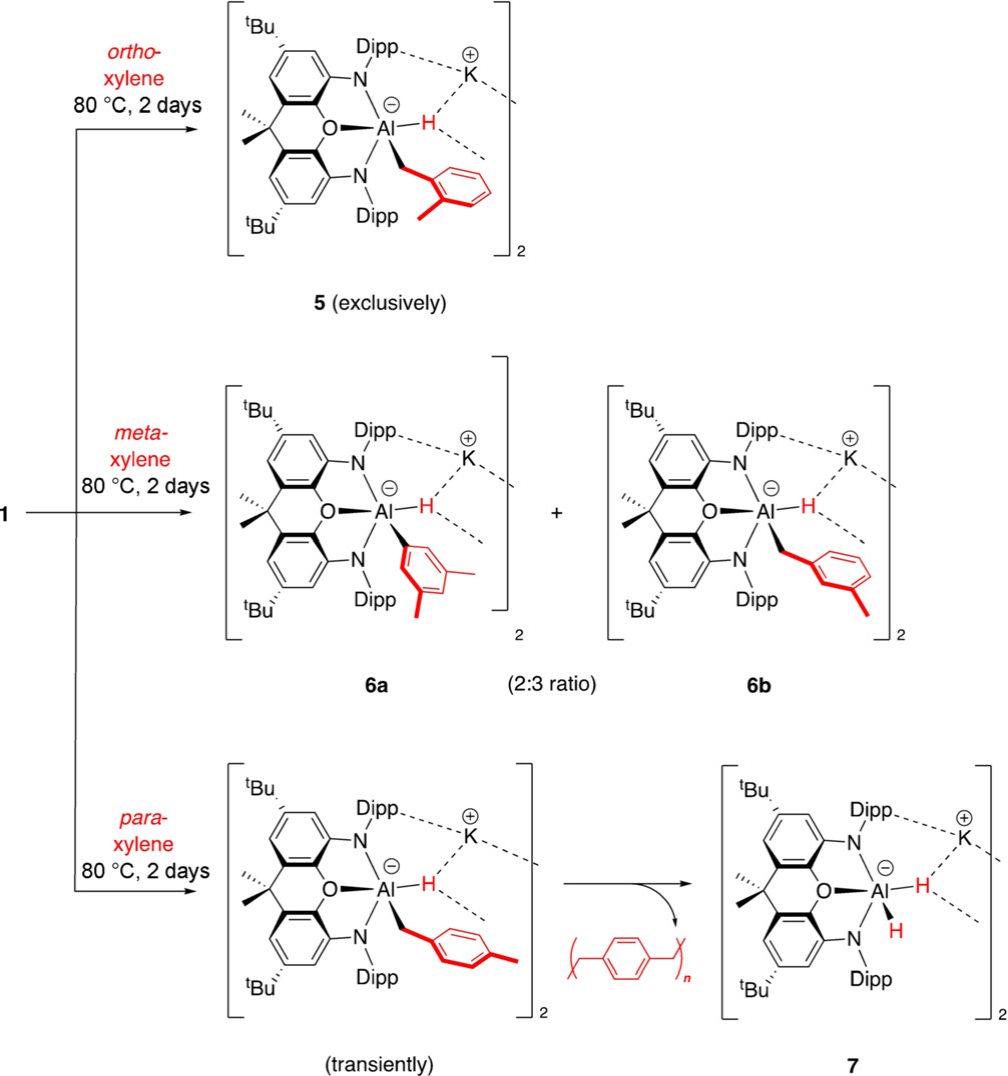
Reactions of **1** with xylenes: benzylic and *meta*‐C−H activation with *ortho*‐ and *meta*‐xylenes; benzylic C−H activation and elimination of *para*‐xylylene with *para*‐xylene.

To rationalise the observed *meta* selectivity in aryl C−H activation we sought a system which we could examine (both experimentally and computationally) that exclusively undergoes *arene* C−H activation, with no competing reactivity at the benzylic positions. We therefore examined the reaction of **1** with *n*‐butylbenzene, hypothesising that the more sterically encumbered and less acidic nature of the benzylic C−H bonds in this system might bias the product mixture further in favour of aryl C−H activation (cf. toluene). At 80 °C this reaction proceeds more slowly than with toluene (taking 7 days to reach completion), but it does indeed lead to exclusive activation of the *meta*‐C−H bonds (within the limits of detection of ^1^H NMR spectroscopy; Scheme 5). As such, the only product observed (either in situ or in the isolated product) is K_2_[(NON)AlH(C_6_H_4_^*n*^Bu‐3)]_2_ (**8**), the structure of which has also been confirmed crystallographically.

**Scheme 5:**
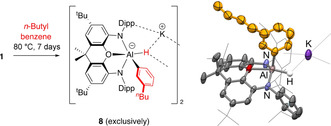
Left: Reaction of **1** with *n*‐butylbenzene. Right: Molecular structure of **8** within the dimeric unit as determined by X‐ray crystallography. Thermal ellipsoids set at the 50 % probability level. H atoms omitted and selected groups shown in wireframe format for clarity. Selected bond lengths [Å] and angles [°]: Al–O 2.127(3), Al–N 1.934(6), 1.940(4), Al–C 2.003(4), Al–H 1.7(1); N‐Al‐N 129.6(2).^[15]^

Acknowledging the more sterically protected nature of the *ortho*‐C−H positions, we were interested to probe the underlying reasons for the observed selectivity, and in particular for the unusual *meta* versus *para* discrimination. In previous work we showed by quantum chemical methods that C−H activation in benzene (Scheme 1) proceeds via nucleophilic attack by the aluminyl, generating a Meisenheimer type transition state which collapses by C‐to‐Al hydride migration.[^11e^, ^16^, ^17^] A similar concerted S_N_Ar (cS_N_Ar) mechanism has also been advanced by Fernandez and Cabrera‐Trujillo for arene C−H activation in benzene and extended arenes by aluminyl reagents.[^18^, ^19^, ^20^] That said, the relatively close approach of the H atom of the C−H bond to the aluminium centre in the transition state (ca. 1.8–1.9 Å)[^16^, ^17^] also suggests analogies with a classical oxidative addition pathway featuring a three‐centre interaction.^[21]^

With this in mind, we exploited density functional theory to probe the relative energies of the transition states corresponding to *ortho*, *meta* and *para* arene‐C−H activation in *n*‐butylbenzene by **1′** (i.e. a cut‐down monomeric aluminyl system [(NON′)Al]^−^ featuring methyl groups in the 2‐ and 7‐positions of the NON ligand, rather than ^*t*^Bu). Notwithstanding the simplifications implied by this (monometallic) system, these calculations reveal not only that C−H insertion is strongly exergonic (to the tune of −145.5, −148.6 and −151.7 kJ mol^−1^ for the *ortho*, *meta* and *para* isomers, respectively), but also that the kinetic barriers associated with activation of the C−H bonds of *n*‐butylbenzene decrease in the order *para* (+125.7 kJ mol^−1^) > *ortho* (+124.7 kJ mol^−1^) > *meta* (+121.9 kJ mol^−1^). Similar transition state energies are calculated for the activation of the arene C−H bonds in toluene (+133.9, +130.4, +126.0 kJ mol^−1^ for *para*, *ortho* and *meta* activation).^[22]^

The origins of the lower energetic barrier in the case of the *meta* C−H activation pathway can, in turn, be rationalised on the basis of resonance theory (Figure 3).^[23]^ The fact that the transition state resembles a classical Meisenheimer complex, implies that the negative charge on the carbocyclic ring is carried to a greater degree by the positions *ortho* and *para* to the entering aluminyl nucleophile. As such, the location of electron‐donating substituents (e.g. Me or ^*n*^Bu) in these positions is disfavoured. This hypothesis is corroborated by an analysis of the NPA charges at the carbon atoms of ring for the corresponding Meisenheimer transition state in the case of benzene. Charges of −0.215/−0.214 are calculated for the two *meta* positions, as opposed to −0.342/−0.371 for the *ortho* carbons, and −0.433 for the *para* position. It is interesting to note, with this in mind, that Harder has recently reported double C−H activation in benzene by an aluminyl system in which the two aluminium fragments are arranged in mutually *para* positions. In this case however, the orientation of the second C−H activation event is ascribed to a templation effect. In our systems we see no evidence for multiple C−H activation events within the same substrate molecule.^[11e]^

**Figure 3:**
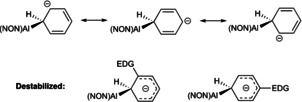
Resonance structures relevant to the Meisenheimer‐type transition state for arene C−H activation by **1** (EDG=electron‐donating group).

In summary, arene C−H activation chemistry by an electron rich Al^I^ compound proceeds via a mechanism involving nucleophilic attack on the aromatic ring. The observed selectivity for *meta*‐C−H attack can be rationalised on the basis of the charge distribution in a transition state which resembles a classical Meisenheimer complex.

## Conflict of interest

The authors declare no conflict of interest.

## Supporting information

As a service to our authors and readers, this journal provides supporting information supplied by the authors. Such materials are peer reviewed and may be re‐organized for online delivery, but are not copy‐edited or typeset. Technical support issues arising from supporting information (other than missing files) should be addressed to the authors.

Supplementary
