# Supplementary material for: Arene C−H Activation at Aluminium(I): meta Selectivity Driven by the Electronics of SNAr Chemistry
Source: Angew Chem Int Ed Engl. 2020 Sep 2;59(46):20376–80. doi: 10.1002/anie.202008557 (PMC7693242; doi:10.1002/anie.202008557)
Supplement: Supplementary file 1 — Supplementary [file ANIE-59-20376-s001.pdf]

Angewandte Chemie
Eine Zeitschrift der Gesellschaft Deutscher Chemiker

Supporting Information

Arene C–H Activation at Aluminium(I): *meta* Selectivity Driven by the Electronics of S_NAr Chemistry

Jamie Hicks, Petra Vasko, Andreas Heilmann, Jose M. Goicoechea, and Simon Aldridge**

anie_202008557_sm_miscellaneous_information.pdf

Supplementary Information

This PDF file includes:

Materials and Methods s2

Synthetic and characterizing data for new compounds s3

^{1}H NMR spectra of new compounds s7

X-ray crystallographic studies s10

Computational details s18

References for supporting information s36

Figures S1 – S12

Table S1

(36 pages total)

Materials and Methods

General considerations. All manipulations were carried out using standard Schlenk line or dry-box techniques under an atmosphere of argon or dinitrogen. Solvents were degassed by sparging with argon and dried by passing through a column of the appropriate drying agent. Xylenes (ortho, meta and para) and *n*-butylbenzene were refluxed over potassium, with the solvent then being distilled and stored under argon in Teflon valve ampoules. NMR spectra were measured in benzene-d_6 (which was dried over potassium) or thf-d_8 (which was dried over $LiAlH_4$), with the solvent then being distilled under reduced pressure and stored under argon in Teflon valve ampoules. NMR samples were prepared under argon in 5 mm Wilmad 507-PP tubes fitted with J. Young Teflon valves. ^1H and $^{13}C\{^1H\}$ NMR spectra were recorded on Bruker Avance III HD nanobay 400 MHz or Bruker Avance III 500 MHz spectrometer at ambient temperature and referenced internally to residual protio-solvent (^1H) or solvent (^{13}C) resonances and are reported relative to tetramethylsilane (δ = 0 ppm). Assignments were confirmed using two-dimensional ^1H-^1H and ^{13}C-^1H NMR correlation experiments. Chemical shifts are quoted in δ (ppm) and coupling constants in Hz. Elemental analyses were carried out by London Metropolitan University. Compound **1** was prepared by the literature method.[S1]

Synthetic and characterizing data for new compounds

Toluene activation – synthesis of 3a and 3b. A solution of **1** (0.200 g, 0.136 mmol) in toluene (10 mL) was heated at 80 °C and stirred for 2 days. During the 2 day reaction, the solution slowly changed colour from yellow to almost colourless. The reaction was allowed to slowly cool to room temperature and left to stand at room temperature overnight to give crystals containing a mixture of **3a** and **3b** in an approximate 2:1 ratio (0.180 g, 80%). **3a**; ^{1}H NMR (500 MHz, d$_8$-THF, 298 K): δ = 0.22 (d, $^{3}J_{HH}$ = 6.7 Hz, 12H, CH(C*H*$_3$)$_2$), 0.74 (d, $^{3}J_{HH}$ = 6.9 Hz, 12H, CH(C*H*$_3$)$_2$), 0.96 (d, $^{3}J_{HH}$ = 6.8 Hz, 12H, CH(C*H*$_3$)$_2$), 1.11 (s, 36H, C(C*H*$_3$)$_3$), 1.26 (d, $^{3}J_{HH}$ = 6.9 Hz, 12H, CH(C*H*$_3$)$_2$), 1.70 (s, 6H, C(C*H*$_3$)$_2$), 1.83 (s, 6H, C(C*H*$_3$)$_2$), 2.12 (s, 6H, Al-tol *m*-C*H*$_3$), 3.05 (sept., $^{3}J_{HH}$ = 6.8 Hz, 4H, C*H*(CH$_3$)$_2$), 3.60 (br., 2H, Al*H*), 3.71 (sept., $^{3}J_{HH}$ = 6.8 Hz, 4H, C*H*(CH$_3$)$_2$), 5.66 (d, 4H, $^{4}J_{HH}$ = 1.9 Hz, NON-*o*-C*H*), 6.43 (d, $^{4}J_{HH}$ = 1.9 Hz, 4H, NON-*p*-C*H*), 6.73 (m, 2H, Al-tol *p*-C*H*), 6.86 (m, 2H, Al-tol *m*-C*H*), 6.98-7.23 (m, 12H Ar*H*), 7.33 (br., 4H, Al-tol *o*-C*H*); ^{13}C{^{1}H} NMR (126 MHz, d$_8$-THF, 298 K): δ = 21.9 (Al-tol *C*H$_3$) 23.2 (C(*C*H$_3$)$_2$), 23.8, 24.8, 25.8, 26.4 (CH(*C*H$_3$)$_2$), 27.9, 28.8 (*C*H(CH$_3$)$_2$), 32.0 (C(*C*H$_3$)$_3$), 33.9 (C(*C*H$_3$)$_2$), 35.2 (*C*(CH$_3$)$_3$), 36.6 (*C*(CH$_3$)$_2$), 103.4, 108.7, 123.2, 124.3, 124.6, 125.9 126.7, 130.9, 134.6, 135.2, 139.0, 140.3, 145.4, 146.3, 146.4, 148.6, 150.2 (Ar-*C*), 159.7 (Al-*C*): **3b**; ^{1}H NMR (500 MHz, d$_8$-THF, 298 K): δ = 0.93 (d, $^{3}J_{HH}$ = 6.8 Hz, 12H, CH(C*H*$_3$)$_2$), 0.98 (d, $^{3}J_{HH}$ = 6.8 Hz, 12H, CH(C*H*$_3$)$_2$), 1.00 (d, $^{3}J_{HH}$ = 6.8 Hz, 12H, CH(C*H*$_3$)$_2$), 1.09 (s, 36H, C(C*H*$_3$)$_3$), 1.26 (d, $^{3}J_{HH}$ = 6.8 Hz, 12H, CH(C*H*$_3$)$_2$), 1.17 (s, 4H, AlC*H*$_2$), 1.52 (s, 6H, C(C*H*$_3$)$_2$), 1.62 (s, 6H, C(C*H*$_3$)$_2$), 3.60 (br., 2H, Al*H*), 3.67 (sept., $^{3}J_{HH}$ = 6.8 Hz, 4H, C*H*(CH$_3$)$_2$), 3.72 (sept., $^{3}J_{HH}$ = 6.8 Hz, 4H, C*H*(CH$_3$)$_2$), 5.64 (d, 4H, $^{4}J_{HH}$ = 2.0 Hz, NON-*o*-C*H*), 5.98 (m, 4H, Al-Bz *o*-C*H*), 6.31 (m, 2H Al-Bz *p*-CH), 6.32 (d, $^{4}J_{HH}$ = 2.0 Hz, 4H, NON-*p*-C*H*), 6.53 (m, 4H, Al-Bz *m*-C*H*), 6.98-7.23 (m, 12H Ar*H*); ^{13}C{^{1}H} NMR (126 MHz, d$_8$-THF, 298 K): δ = 24.8, 25.1, 25.6, 25.8 (CH(*C*H$_3$)$_2$), 26.0 (C(*C*H$_3$)$_2$), 26.7 (Al*C*H$_2$), 28.2, 28.3 (*C*H(CH$_3$)$_2$), 31.5 (C(*C*H$_3$)$_2$), 32.1 (C(*C*H$_3$)$_3$), 35.1 (*C*(CH$_3$)$_3$), 36.5 (*C*(CH$_3$)$_2$), 103.0, 109.0, 118.1, 123.6, 124.2, 124.4, 126.8, 128.2, 130.5, 146.1, 146.3, 147.7, 148.3, 148.5, 154.2 (Ar-*C*). anal. calc. for $C_{108}H_{140}Al_2K_2N_4O_2$: C 78.21%, H 8.51%, N 3.38%, found: C 78.13%, H 8.64%, N 3.52%.

Anisole activation – synthesis of 4. A solution of **1** (0.200 g, 0.136 mmol) in anisole (10 mL) was heated at 80 °C and stirred for 30 mins. On heating the reaction solution to 80 °C, the solution changed colour from yellow to almost colourless. The reaction was allowed to cool to room temperature whereupon volatiles were removed *in vacuo*. The residual was dissolved in the minimum volume of warm toluene (*ca.* 2 mL at 60 °C) and slowly cooled to room temperature overnight to give **4** as colourless crystals (0.190 g, 83%). ^{1}H NMR (500 MHz, C$_6$D$_6$, 298 K): δ = 0.10 (br., 6H, AlC*H*$_3$) 1.13-1.19 (m, 48H, CH(C*H*$_3$)$_2$), 1.34 (s, 36H, C(C*H*$_3$)$_3$), 1.60 (s, 12H, C(C*H*$_3$)$_2$), 3.93 (br., 4H, C*H*(CH$_3$)$_2$), 4.02 (br., 4H, C*H*(CH$_3$)$_2$), 6.03 (d, 4H, $^{4}J_{HH}$ = 1.9 Hz, NON-*o*-C*H*), 6.30 (br., 4H, OPh *o*-C*H*), 6.51 (br., 2H, OPh *p*-C*H*), 6.70 (d, $^{4}J_{HH}$ = 1.9 Hz, 4H, NON-*p*-C*H*), 6.84 (br., 4H, OPh *m*-C*H*), 6.98-7.13 (m, 12H, Ar*H*), ^{13}C{^{1}H} NMR (126 MHz, C$_6$D$_6$, 298 K): δ = –3.7 (Al-*C*H$_3$), 23.5 (C(*C*H$_3$)$_2$), 25.0, 25.5, 25.9, 26.0 (CH(*C*H$_3$)$_2$), 27.8, 28.1 (*C*H(CH$_3$)$_2$), 32.0 (C(*C*H$_3$)$_2$), 32.2 (C(*C*H$_3$)$_3$), 35.2 (*C*(CH$_3$)$_3$), 36.6 (*C*(CH$_3$)$_2$), 105.0, 109.0, 117.8, 120.4, 123.5, 124.3, 124.6, 129.9, 130.6, 139.1, 145.1, 146.7, 147.7, 148.6, 149.9 (Ar-*C*),

161.4 (Al-*C*); anal. calc. for $C_{108}H_{140}Al_2K_2N_4O_4$: C 76.73%, H 8.35%, N 3.31%, found: C 76.67%, H 8.69%, N 3.51%.

***o*-Xylene activation – synthesis of 5.** A solution of **1** (0.200 g, 0.136 mmol) in *o*-xylene (10 mL) was heated and stirred at 80 °C for 2 days. During the 2 day reaction, the solution slowly changed colour from yellow to almost colourless. The reaction was allowed to slowly cool to room temperature overnight to give **5** as colourless crystals (0.155 g, 68%). ^{1}H NMR (500 MHz, d$_8$-THF, 298 K): δ = 0.88 (d, $^3J_{HH}$ = 6.8 Hz, 12H, CH(CH_3)$_2$), 0.92 (d, $^3J_{HH}$ = 6.9 Hz, 12H, CH(CH_3)$_2$), 0.95 (d, $^3J_{HH}$ = 6.8 Hz, 12H, CH(CH_3)$_2$), 1.08 (s, 36H, C(CH_3)$_3$), 1.15 (d, $^3J_{HH}$ = 6.9 Hz, 12H, CH(CH_3)$_2$), 1.26 (s, 4H, AlCH_2), 1.57 (s, 6H, C(CH_3)$_2$), 1.62 (s, 6H, Al-xy *o*-CH_3), 1.69 (s, 6H, C(CH_3)$_2$), 3.58 (sept., $^3J_{HH}$ = 6.8 Hz, 4H, C*H*(CH$_3$)$_2$), 3.68 (sept., $^3J_{HH}$ = 6.8 Hz, 4H, C*H*(CH$_3$)$_2$), 4.30 (br., 2H, Al*H*), 5.61 (d, 4H, $^4J_{HH}$ = 1.9 Hz, NON-*o*-C*H*), 5.90 (m, 2H, Al-xy *o*-C*H*), 6.34 (m, 4H, Al-xy *m*- & *p*-C*H*), 6.55 (m, 2H, Al-xy *m*-C*H*), 7.04-7.19 (m, 12H, Ar*H*); ^{13}C{^{1}H} NMR (126 MHz, d$_8$-THF, 298 K): δ = 21.7 (Al-xy *C*H$_3$) 24.6, 24.7, 25.8, (CH(*C*H$_3$)$_2$), 26.3 (C(*C*H$_3$)$_2$), 26.7 (CH(*C*H$_3$)$_2$), 28.3, 28.5 (*C*H(CH$_3$)$_2$), 32.0 (C(*C*H$_3$)$_3$), 35.1 (*C*(CH$_3$)$_3$), 36.7 (*C*(CH$_3$)$_2$), 103.1, 109.5, 119.0, 123.6, 124.3, 124.7, 128.4, 129.3, 131.0, 134.5, 140.7, 145.6, 146.3, 148.0. 148.5, 148.6, 151.8 (Ar-*C*); anal. calc. for $C_{110}H_{144}Al_2K_2N_4O_2$: C 78.34%, H 8.61%, N 3.32%, found: C 78.50%, H 8.75%, N 3.16%.

***m*-Xylene activation – synthesis of 6a and 6b.** A solution of **1** (0.200 g, 0.136 mmol) in *m*-xylene (10 mL) was heated at 80 °C and stirred for 2 days. During the 2 day reaction, the solution slowly changed colour from yellow to almost colourless. The reaction was allowed to cool to room temperature and volatiles were removed *in vacuo*. The residual was dissolved in the minimum volume of boiling *n*-hexane (*ca.* 5 mL) and slowly cooled to room temperature overnight to give colourless crystals containing a mixture of **6a** and **6b** in an approximate 1:2 ratio (0.160 g, 70%). **6a**; ^{1}H NMR (500 MHz, d$_8$-THF, 298 K): δ = 0.21 (d, $^3J_{HH}$ = 6.7 Hz, 12H, CH(CH_3)$_2$), 0.74 (d, $^3J_{HH}$ = 6.9 Hz, 12H, CH(CH_3)$_2$), 0.96 (d, $^3J_{HH}$ = 6.8 Hz, 12H, CH(CH_3)$_2$), 1.11 (s, 36H, C(CH_3)$_3$), 1.26 (d, $^3J_{HH}$ = 6.9 Hz, 12H, CH(CH_3)$_2$), 1.70 (s, 6H, C(CH_3)$_2$), 1.83 (s, 6H, C(CH_3)$_2$), 2.10, (s, 6H, m-CH_3), 2.13 (s, 6H, m-CH_3), 3.04 (sept., $^3J_{HH}$ = 6.8 Hz, 4H, C*H*(CH$_3$)$_2$), 3.67 (sept., $^3J_{HH}$ = 6.9 Hz, 4H, C*H*(CH$_3$)$_2$), 3.80 (br., 2H, Al*H*), 5.67 (d, 4H, $^4J_{HH}$ = 1.9 Hz, NON-*o*-C*H*), 6.43 (d, $^4J_{HH}$ = 1.9 Hz, 4H, NON-*p*-C*H*), 6.73 (m, 2H, Al-xy *p*-C*H*), 6.98-7.18 (m, 12H Ar*H*), 7.25 (br., 4H, Al-xy *o*-C*H*); ^{13}C{^{1}H} NMR (126 MHz, d$_8$-THF, 298 K): δ = 21.8, 21.9 (Al-xy *C*H$_3$) 23.2 (C(*C*H$_3$)$_2$), 23.8, 24.9, 25.8, 26.8 (CH(*C*H$_3$)$_2$), 27.9, 28.8 (*C*H(CH$_3$)$_2$), 32.0 (C(*C*H$_3$)$_3$), 33.9 (C(*C*H$_3$)$_2$), 35.2 (*C*(CH$_3$)$_3$), 36.6 (*C*(CH$_3$)$_2$), 103.3, 108.7, 123.2, 124.3, 124.6, 125.2, 125.9 126.8, 130.7, 134.6, 135.2, 139.0, 140.2, 145.4, 146.5, 148.5, 148.7, 150.1 (Ar-*C*), 159.6 (Al-*C*); **6b**; ^{1}H NMR (500 MHz, d$_8$-THF, 298 K): δ = 0.93 (d, $^3J_{HH}$ = 6.8 Hz, 12H, CH(CH_3)$_2$), 0.99 (d, $^3J_{HH}$ = 6.9 Hz, 12H, CH(CH_3)$_2$), 1.02 (d, $^3J_{HH}$ = 6.7 Hz, 12H, CH(CH_3)$_2$), 1.10 (s, 36H, C(CH_3)$_3$), 1.15 (s, 4H, AlCH_2), 1.27 (d, $^3J_{HH}$ = 6.9 Hz, 12H, CH(CH_3)$_2$), 1.52 (s, 6H, C(CH_3)$_2$), 1.58 (s, 6H, C(CH_3)$_2$), 1.89 (s, 6H, Al-xy *m*-CH$_3$), 3.70 (sept., $^4J_{HH}$ = 1.9 Hz, 8H, C*H*(CH$_3$)$_2$), 3.80 (br., 2H, Al*H*), 5.65 (d, $^4J_{HH}$ = 1.9 Hz, 4H, NON-*o*-C*H*), 5.66 (br., 2H, Al-xy AlCH$_2$CC*H*CMe), 5.95 (d, $^3J_{HH}$ = 7.6 Hz, 2H, Al-xy AlCH$_2$CC*H*CH), 6.16 (d, $^3J_{HH}$ = 7.5 Hz, 2H, Al-xy *p*-CH), 6.31 (d, $^4J_{HH}$ = 1.9 Hz, 4H, NON-*p*-C*H*), 6.47 (t, $^4J_{HH}$ = 7.5

Hz, 2H, Al-xy *m*-C*H*), 6.98-7.24 (m, 12H, Ar*H*); $^{13}C\{^1H\}$ NMR (126 MHz, d_8-THF, 298 K): δ = 21.6 (Al-xy *m*-CH_3), 24.2, 24.6, 25.6 (CH(CH_3)$_2$), 25.8 (C(CH_3)$_2$), 26.4 (CH(CH_3)$_2$), 26.4 (AlCH_2), 28.2, 28.3 (CH(CH_3)$_2$), 31.3 (C(CH_3)$_2$), 32.1 (C(CH_3)$_3$), 35.1 (C(CH_3)$_3$), 36.5 (C(CH_3)$_2$), 103.0, 108.9, 119.1, 123.6, 124.1, 124.3, 124.4, 126.5, 127.9, 130.4, 130.8, 135.4, 140.3, 145.7, 146.0, 146.2, 148.3, 148.5, 153.9 (Ar-*C*); anal. calc. for $C_{110}H_{144}Al_2K_2N_4O_2$: C 78.34%, H 8.61%, N 3.32%, found: C 78.21%, H 8.72%, N 3.17%.

***p*-Xylene activation.** A solution of **1** (0.200 g, 0.136 mmol) in *p*-xylene (10 mL) was heated at 80 °C and stirred for 2 days. During the 2 day reaction, the solution slowly changed colour from yellow to almost colourless. However, unlike the reactions with ortho- and meta-xylene, this reaction produced a sticky colourless polymer-like precipitate. Analysis of a ^1H NMR spectrum taken of the crude reaction mixture revealed two major **NON** containing products in an approximate 60:40 ratio. The resonances associated with the major of these species match perfectly with those of the previously reported dihydride complex **7**.[S1] The resonances of the other species are consistent with C–H activation of a methyl group of *p*-xylene, as these closely resemble those of **3b**. These observations suggest that the C–H activation of *p*-xylene occurs on a methyl group to give the corresponding anion [(**NON**)AlH{CH_2(4-Me-C_6H_4)]$^-$. Under the reaction conditions however, this anion eliminates *p*-xylylene (which readily polymerises to give poly(para-xylene)) yielding the aluminium dihydride anion [(**NON**)AlH_2]$^-$. Attempts to slow down/stop the elimination of *p*-xylylene from the anion were attempted (by lowering the temperature of the reaction whilst also adjusting the reaction time). However, after multiple attempts, the best isolated ratio of [(**NON**)AlH{CH_2(4-Me-C_6H_4)]$^-$ to [(**NON**)AlH_2]$^-$ was 70:30. This was achieved by heating a solution of K_2[(**NON**)Al]$_2$ (200 mg) in *p*-xylene (10 mL) to 75 °C for 18 hours. Attempts to purify this reaction mixture by crystallization were unfortunately also unsuccessful, as K_2[(**NON**)AlH_2]$_2$ **7** seemed to crystallize preferentially to K_2[(**NON**)AlH{CH_2(4-Me-C_6H_4)]. However, one reaction produced a handful of crystals of the mixed anion dimer K_2[(**NON**)AlH{CH_2(4-Me-C_6H_4)][(**NON**)AlH_2] **9**.

***n*-Butylbenzene activation – synthesis of 8.** A suspension of K_2[(**NON**)Al]$_2$ (0.200 g, 0.136 mmol) in *n*-butylbenzene (10 mL) was heated and stirred at 80 °C for 7 days. During the 7 day reaction, the reaction mixture slowly changed from a yellow solution/suspension to an almost colourless solution. The reaction was allowed to slowly cool to room temperature, concentrated *in vacuo* (*ca.* 2 mL) and left to stand at room temperature overnight to give **8** as colourless crystals (0.128 g, 54%). ^1H NMR (400 MHz, C_6D_6, 298 K): δ = 0.48 (d, $^3J_{HH}$ = 6.6 Hz, 12H, CH(CH_3)$_2$), 0.83 (t, $^3J_{HH}$ = 7.2 Hz, 6H, $CH_2CH_2CH_2$CH_3), 0.95 (d, $^3J_{HH}$ = 6.8 Hz, 12H, CH(CH_3)$_2$), 1.09 (d, $^3J_{HH}$ = 6.7 Hz, 12H, CH(CH_3)$_2$), 1.25 (d, $^3J_{HH}$ = 6.7 Hz, 12H, CH(CH_3)$_2$), 1.27 (s, 36H, C(CH_3)$_3$), 1.29 (m, 4H, CH_2CH_2C$H_2$$CH_3$), 1.66 (m, 4H, CH_2C$H_2$$CH_2CH_3$), 1.71 (s, 6H, C(C$H_3$)$_2$), 1.91 (s, 6H, C(C$H_3$)$_2$), 2.72 (t, $^3J_{HH}$ = 7.5 Hz, 4H, C$H_2$$CH_2CH_2CH_3$), 3.31 (sept., $^3J_{HH}$ = 6.7 Hz, 4H, C*H*(CH_3)$_2$), 3.65 (br., 2H, Al*H*), 3.78 (sept., $^3J_{HH}$ = 6.7 Hz, 4H, C*H*(CH_3)$_2$), 5.83 (s, 4H, NON-*o*-C*H*), 6.77 (s, 4H, NON-*p*-C*H*), 6.95 (br., 2H, AlCC*H*CH), 7.01-7.22 (m, 14H Ar*H*), 7.36 (br., 2H, AlCCHC*H*), 8.18 (br., 2H, AlCC*H*); $^{13}C\{^1H\}$ NMR (126 MHz, C_6D_6): δ = 14.2

($CH_2CH_2CH_2\mathit{C}H_3$), 22.8 ($C(\mathit{C}H_3)_2$), 23.0 ($CH_2CH_2\mathit{C}H_2CH_3$), 25.0, 25.1, 25.5, 26.0 ($CH(\mathit{C}H_3)_2$), 27.6, 28.8 ($\mathit{C}H(CH_3)_2$), 32.0 ($C(\mathit{C}H_3)_3$), 33.8 ($C(\mathit{C}H_3)_2$), 34.6 ($CH_2\mathit{C}H_2CH_2CH_3$), 35.1 ($\mathit{C}(CH_3)_3$), 36.8 ($\mathit{C}(CH_3)_2$), 36.9 ($\mathit{C}H_2CH_2CH_2CH_3$), 105.8, 109.4, 123.8, 125.9, 126.0, 126.7, 126.9, 131.6, 133.0, 139.2, 140.1, 142.0, 144.4, 146.3, 147.0, 149.0, 151.1 (Ar-*C*), 157.4 (Al-*C*); anal. calc. for $C_{114}H_{152}Al_2K_2N_4O_2$: C 78.57%, H 8.79%, N 3.22%, found: C 78.45%, H 8.87%, N 4.34%.

Bromobenzene activation: synthesis of K_2[(NON)AlBr(Ph)]$_2$. To a solution of K_2[(**NON**)Al]$_2$ (0.200 mg, 0.136 mmol) in toluene (10 mL) was added bromobenzene (57 µl, 0.54 mmol). The mixture was stirred for 5 min and then left to stand overnight. The product K_2[(**NON**)AlBr(Ph)]$_2$ was obtained as a white powder, isolated by filtration and dried *in vacuo.* Crystals suitable for X-ray diffraction were obtained from a saturated benzene solution (0.040 g, 17%; > 90% conversion by *in situ* ^{1}H NMR). ^{1}H NMR (400 MHz, d$_8$-THF, 298 K): δ = 0.81 – 0.91 (m, 36H, $CH(C\mathit{H}_3)_2$), 1.11 (s, 36H, $C(C\mathit{H}_3)_3$), 1.15 (d, $^3J_{HH}$ = 6.7 Hz, 12H, $CH(C\mathit{H}_3)_2$), 3.32 (sept, $^3J_{HH}$ = 6.7 Hz, 4H, $C\mathit{H}(CH_3)_2$), 3.67 (sept, $^3J_{HH}$ = 6.7 Hz, 4H, $C\mathit{H}(CH_3)_2$), 5.75 (d, $^4J_{HH}$ = 1.9 Hz, 4H, XA-*o*-C*H*), 6.64 (d, $^4J_{HH}$ = 1.9 Hz, 4H, XA-*p*-C*H*), 6.96 – 7.09 (m, 6H, Ph-Ar*H*), 7.17 (s, 12H, Dipp-Ar*H*), 7.60 (s, 4H, Ph-Ar*H*); ^{13}C NMR (101 MHz, THF-d$_8$): δ = 24.8 (XA-$C(\mathit{C}H_3)_2$), 24.3 ($CH(\mathit{C}H_3)_2$), 25.3 ($CH(\mathit{C}H_3)_2$), 26.7 ($CH(\mathit{C}H_3)_2$), 26.8 ($CH(\mathit{C}H_3)_2$), 28.3 ($\mathit{C}H(CH_3)_2$), 29.4 ($\mathit{C}H(CH_3)_2$), 32.2 ($C(\mathit{C}H_3)_3$), 34.6 (XA-$C(\mathit{C}H_3)_2$), 35.7 ($\mathit{C}(CH_3)_3$), 37.2 (XA-$\mathit{C}(CH_3)_2$), 106.9 (XA-$(CH_3)_2CC\mathit{C}H$), 111.5 (H*C*CN), 125.0 (Ph-*C*H), 125.4 (Dipp-*C*H), 126.8 (Ph-*C*H), 126.9 (Dipp-*C*H), 131.0 (XA-$(CH_3)_2C\mathit{C}$), 138.9 (*C*O), 138.6 (Ph-*C*), 139.5 (Ph-*C*H), 145.1 (N*C*CO), 145.6 (Dipp-*i*-*C*), 147.7 ($\mathit{C}C(CH_3)_3$), 147.9 (Dipp-*m*-*C*), 149.3 (Dipp-*m*-*C*).

^{1}H NMR spectra of new compounds

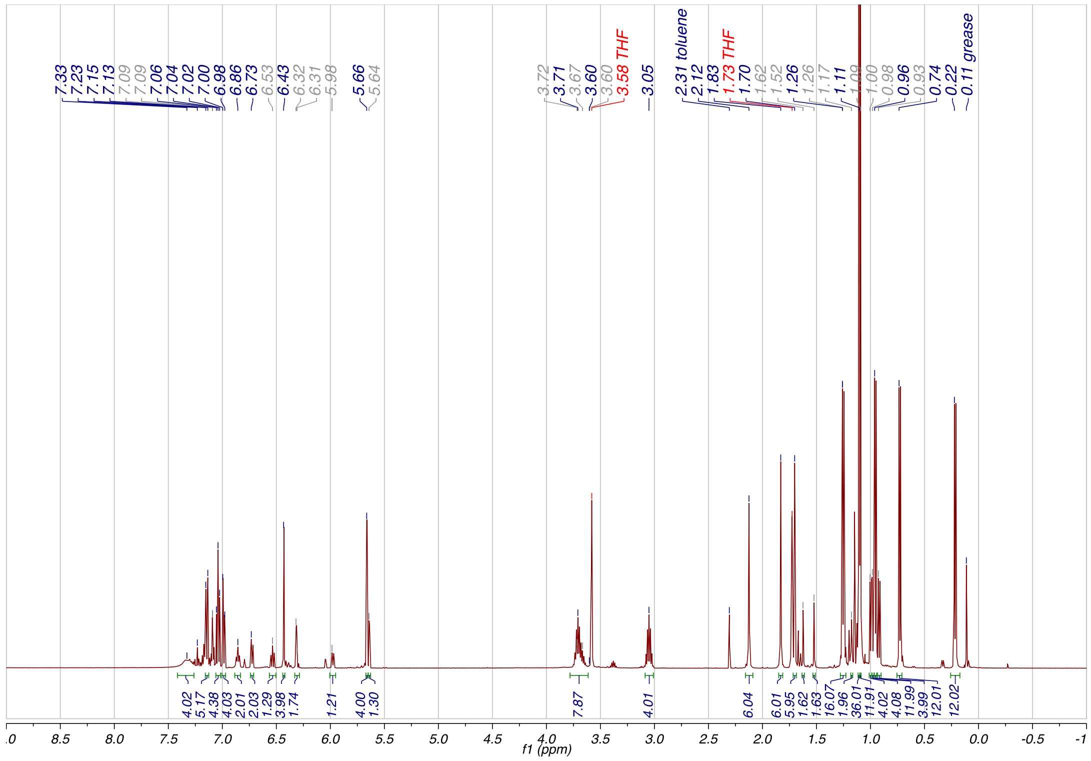

Figure S1. ^{1}H NMR spectrum of the approx. 3:1 mixture of **3a** (peaks marked in blue) and **3b** (peaks marked in gray) in d_8-THF. Resonances corresponding to residual solvent signals and other impurities are marked.

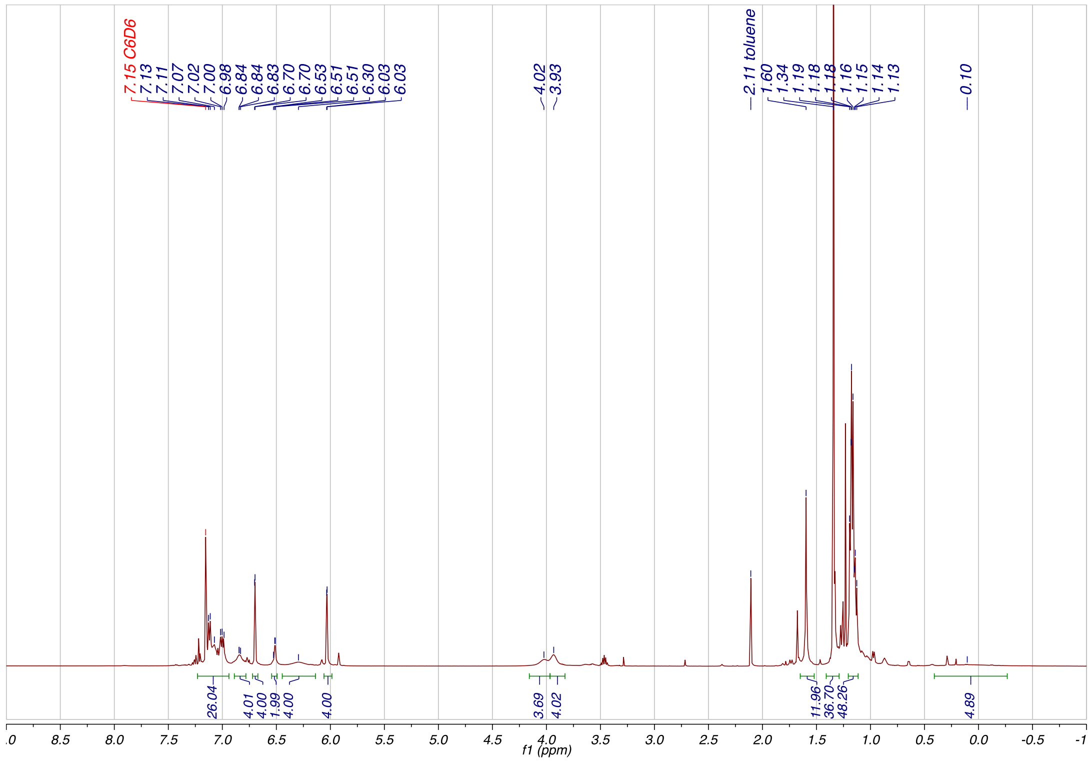

Figure S2. ^{1}H NMR spectrum of **4** in C_6D_6. Resonances corresponding to residual solvent signals and other impurities are marked.

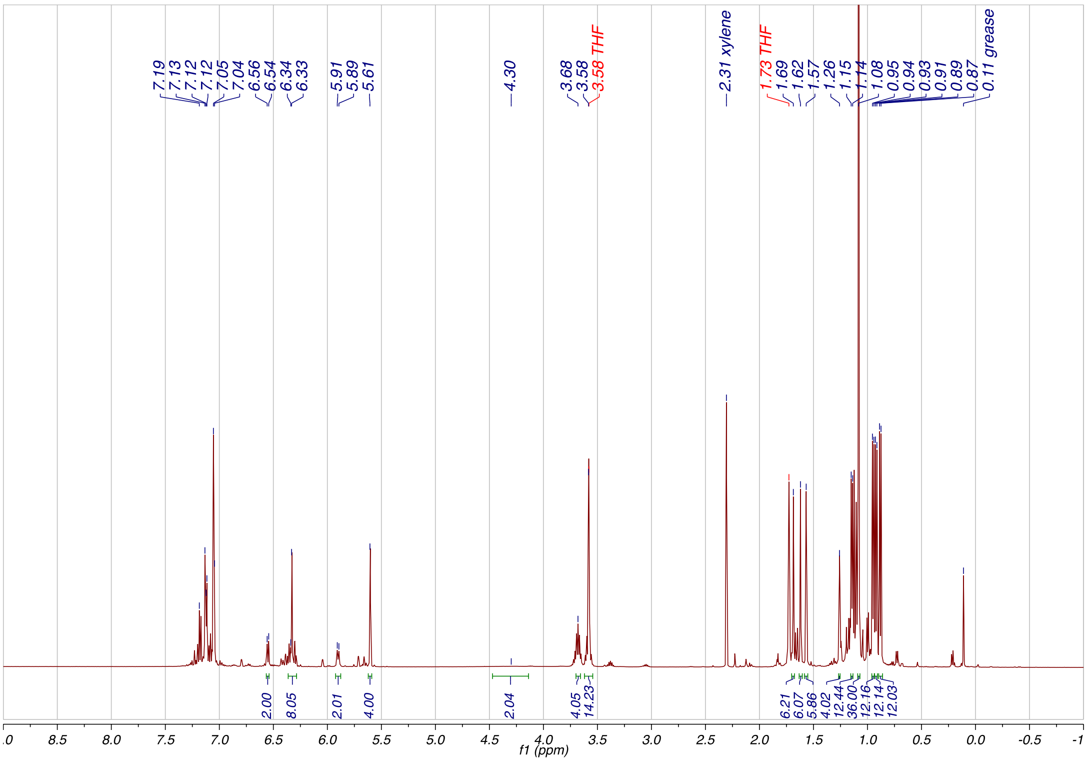

Figure S3. ^{1}H NMR spectrum of **5** in d_8-THF. Resonances corresponding to residual solvent signals and other impurities are marked.

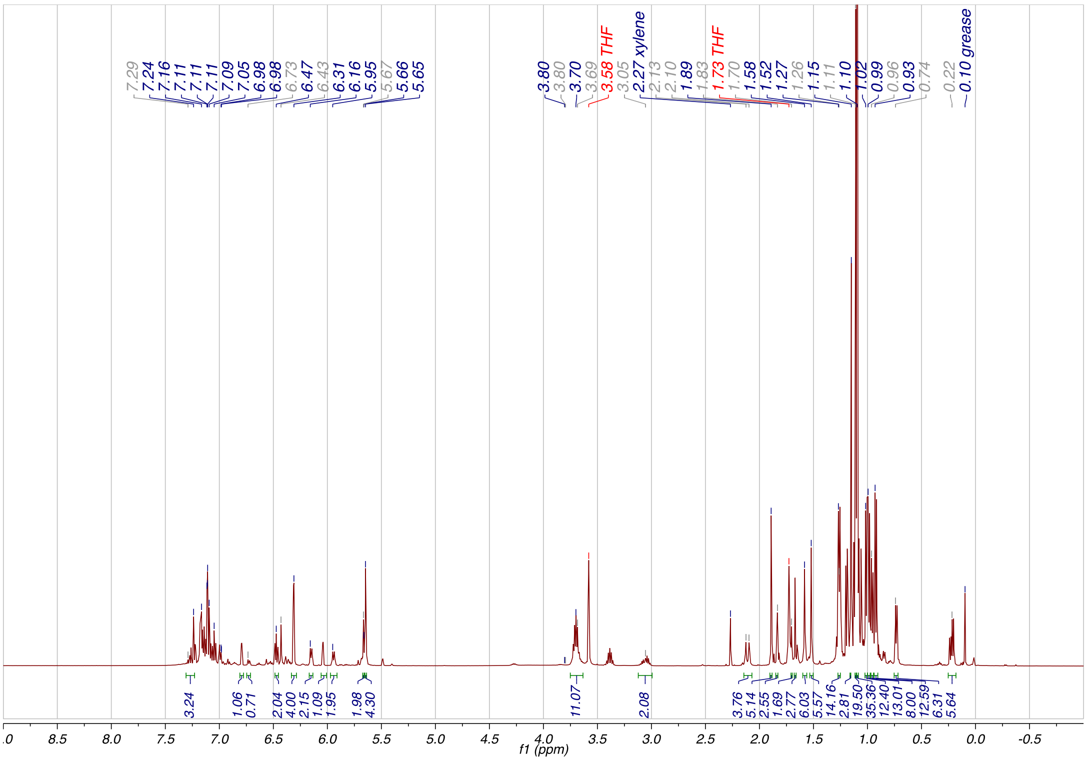

Figure S4. ^{1}H NMR spectrum of an approximate 1:2 mixture of **6a** (peaks marked in grey) and **6b** (peaks marked in blue) in d_8-THF. Resonances corresponding to residual solvent signals and other impurities are marked.

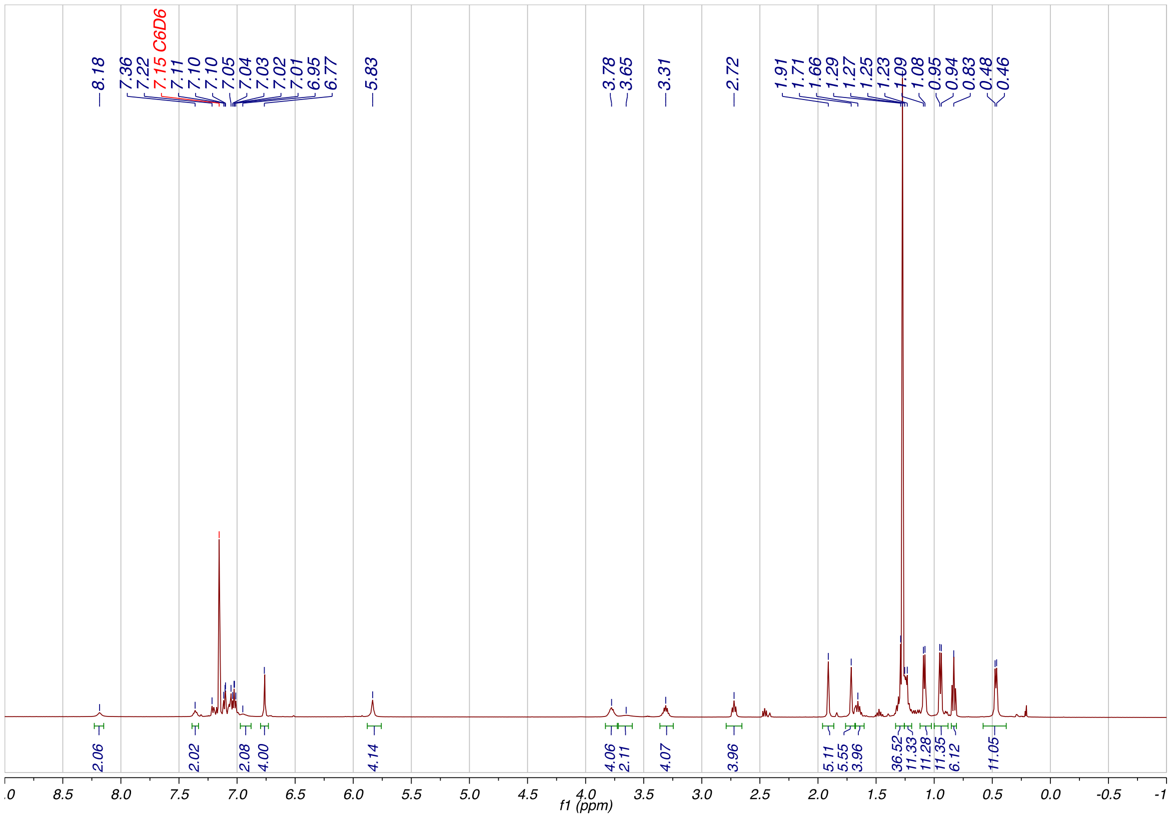

Figure S5. ^{1}H NMR spectrum of **8**. Resonances corresponding to residual solvent signals and other impurities are marked.

X-ray crystallographic studies

Single-crystal X-ray diffraction data were collected using an Oxford Diffraction Supernova dual-source diffractometer equipped with a 135 mm Atlas CCD area detector. Crystals were selected under Paratone-N oil, mounted on Micromount loops and quench-cooled using an Oxford Cryosystems open flow N_2 cooling device.[S2] Data were collected at 150 K using mirror monochromated Cu K_{α} radiation (λ = 1.5418 Å; Oxford Diffraction Supernova) and were processed using the CrysAlisPro package, including unit cell parameter refinement and inter-frame scaling (which was carried out using SCALE3 ABSPACK within CrysAlisPro).[S3] Equivalent reflections were merged and diffraction patterns processed with the CrysAlisPro suite. Structures were subsequently solved using ShelXT 2018[S4] and refined on F^2 using the ShelXL 2018 package[S4] and XSeed.[S5]

Table S1. Selected X-ray data collection and refinement parameters for **3**·4toluene, **4**·6toluene, **5**·*o*-xylene, **6**·2hexane, **8**·^{n}BuPh, K_2[(**NON**)AlH{$CH_2(C_6H_4Me$-4)}][(**NON**)AlH_2]·hexane and K_2[(**NON**)AlBr(Ph)]$_2$·9benzene.

	**3**·4toluene	**4**·6toluene	**5**·*o*-xylene	**6**·2hexane
formula	$C_{136}H_{172}Al_2K_2N_4O_2$	$C_{150}H_{188}Al_2K_2N_4O_4$	$C_{118}H_{154}Al_2K_2N_4O_2$	$C_{122}H_{172}Al_2K_2N_4O_2$
Fw [g mol^{-1}]	2026.93	2243.19	1792.60	1858.79
crystal system	triclinic	triclinic	monoclinic	triclinic
space group	*P*-1	*P*-1	*C*2/c	*P*-1
a (Å)	14.2315(5)	15.4196(6)	22.5942(3)	14.0210(5)
b (Å)	16.0087(7)	15.9239(6)	16.7146(2)	14.3148(8)
c (Å)	16.0903(7)	16.0232(7)	28.5312(5)	16.2311(6)
α (°)	111.398(4)	106.877(4)	90	87.507(4)
β (°)	109.861(4)	115.990(4)	103.365(2)	70.412(3)
γ (°)	101.192(3)	91.894(3)	90	72.639(4)
V (Å^3)	2990.9(2)	3324.9(3)	10483.1(3)	2923.6(2)
Z	1	1	4	1
radiation, λ (Å)	Cu K_α (1.5418)	Cu K_α (1.5418)	Cu K_α (1.5418)	Cu K_α (1.5418)
T (K)	150(2)	150(2)	150(2)	150(2)
ρ_{calc} (g cm^{-3})	1.125	1.120	1.136	1.056
μ (mm^{-1})	1.233	1.166	1.345	1.218
reflections collected	12371	13754	10929	12045
independent reflections	9938	11433	9077	8574
parameters	763	913	749	749
$R_{(int)}$	0.0458	0.0426	0.0346	0.0414
R1 ($I \geq 2\sigma(I)$/all data)	0.0642/ 0.0780	0.0641/0.0741	0.0485/0.0590	0.0820/0.1068
wR2(F^2) ($I \geq 2\sigma(I)$/all data)	0.1752/ 0.1943	0.1782/0.1921	0.1307/0.1392	0.2417/0.2764
GOF	1.054	1.129	1.072	1.063
CCDC deposition number	2008537	2008541	2008539	2008536

Table S1 (contd). Selected X-ray data collection and refinement parameters for **3**·4toluene, **4**·6toluene, **5**·*o*-xylene, **6**·2hexane, **8**·^{n}BuPh, K_2[(**NON**)AlH{$CH_2(C_6H_4Me$-4)}][(**NON**)AlH_2]·hexane and K_2[(**NON**)AlBr(Ph)]$_2$

	**8**·^{n}BuPh	K_2[(**NON**)AlH{$CH_2(C_6H_4Me$-4)}] [(**NON**)AlH_2]·hexane	K_2[(**NON**)AlBr(Ph)]$_2$ ·9Benzene
formula	$C_{124}H_{166}Al_2K_2N_4O_2$	$C_{108}H_{150}Al_2K_2N_4O_2$	$C_{160}H_{188}Al_2Br_2K_2N_4O_2$
Fw [g mol^{-1}]	1876.76	1668.47	2491.11
crystal system	monoclinic	monoclinic	triclinic
space group	*C*2/*c*	*P*2$_1$/*n*	*P*-1
a (Å)	36.433(4)	14.4832(4)	13.6586(3)
b (Å)	27.8547(6)	33.4706(10)	14.1842(5)
c (Å)	19.911(2)	20.8111(6)	20.8356(6)
α (°)	90	90	94.109(2)
β (°)	142.51(2)	95.553(3)	106.773(2)
γ (°)	90	90	111.112(3)
V (Å^3)	12298(4)	10041.1(5)	3535.1(2)
Z	4	4	1
radiation, λ (Å)	Cu K_α (1.5418)	Cu K_α (1.5418)	Cu K_α (1.5418)
T (K)	150(2)	150(2)	150(2)
ρ_{calc} (g cm^{-3})	1.014	1.104	1.170
μ (mm^{-1})	1.164	1.368	1.772
reflections collected	12715	20756	14638
independent reflections	8314	15530	12431
parameters	900	1199	850
$R_{(int)}$	0.0372	0.0454	0.0363
R1 ($I \geq 2\sigma(I)$/all data)	0.0900/0.1214	0.0637/0.0867	0.0469/0.0566
wR2(F^2) ($I \geq 2\sigma(I)$/all data)	0.2608/0.3082	0.1742/0.1961	0.1233/0.1326
GOF	1.184	1.071	1.075
CCDC deposition number	2008538	2008540	2010397

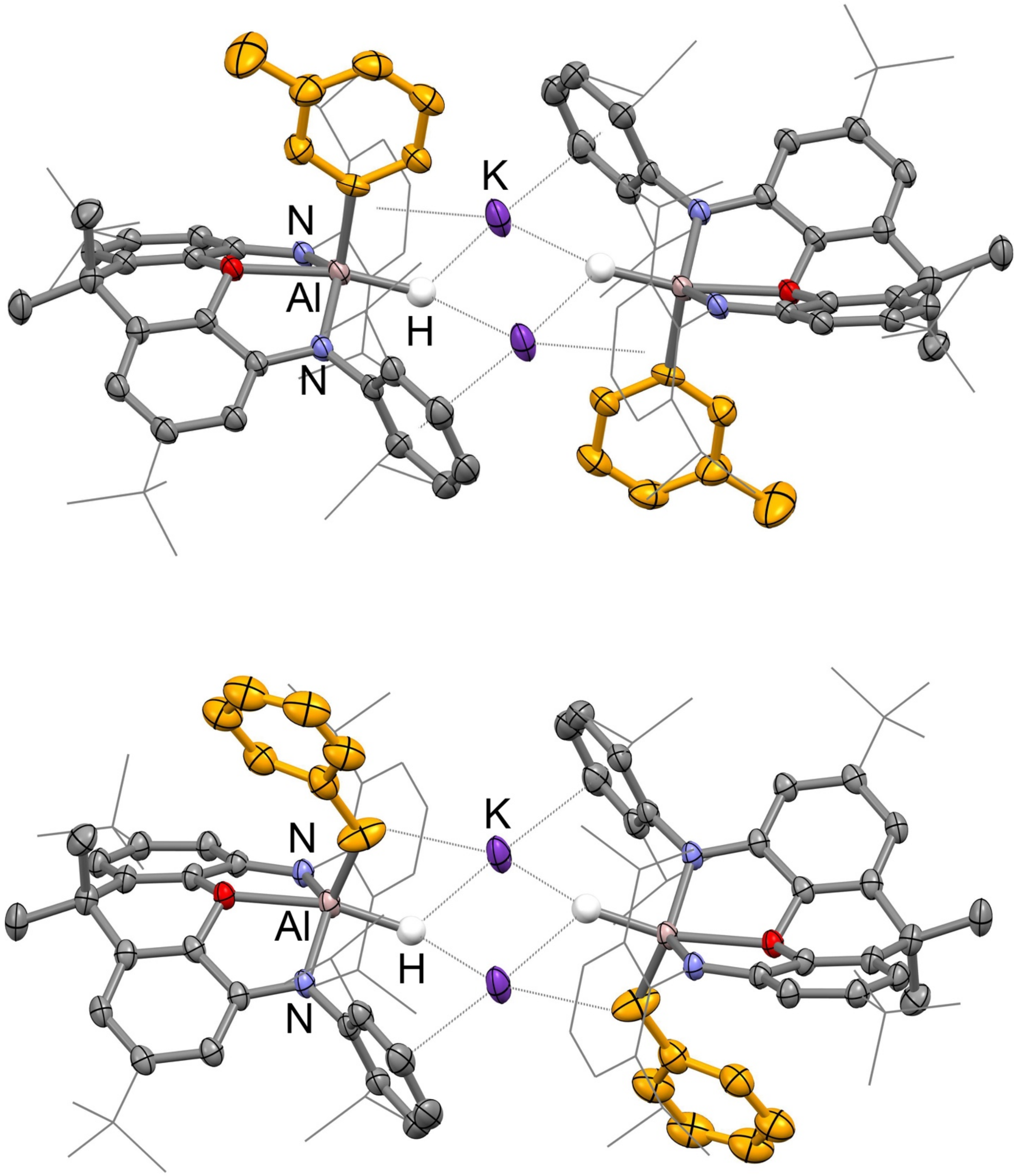

Figure S6. *Molecular structures of **3a** (top) and **3b** (bottom) as determined by X-ray crystallography. Toluene solvate molecules and most hydrogen atoms omitted for clarity. Parts of the ligand are also shown in wireframe for clarity. Thermal ellipsoids set at the 50% probability level. Key bond lengths (Å) and angles (^{o}): Al-N 1.9379(17), 1.9360(16), Al-O 2.1476(13), Al-C 2.047(5), 1.86(2), N-Al-N 129.16(7).*

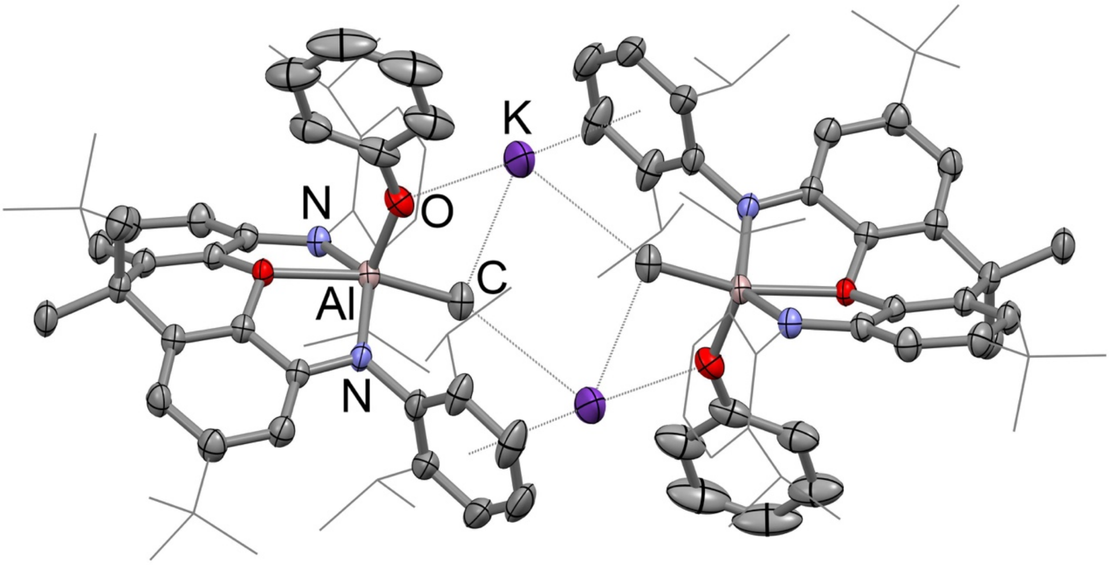

Figure S7. *Molecular structure of **4** as determined by X-ray crystallography. Toluene solvate molecules and hydrogen atoms omitted for clarity. Parts of the ligand are also shown in wireframe for clarity. Thermal ellipsoids set at the 50% probability level. Key bond lengths (Å) and angles (^{o}): Al-N 1.9393(15), 1.9379(14), Al-O(Ph) 1.7975(14), Al-O(NON) 2.1228(11), Al-C 2.0088(19), N-Al-N 134.64(6).*

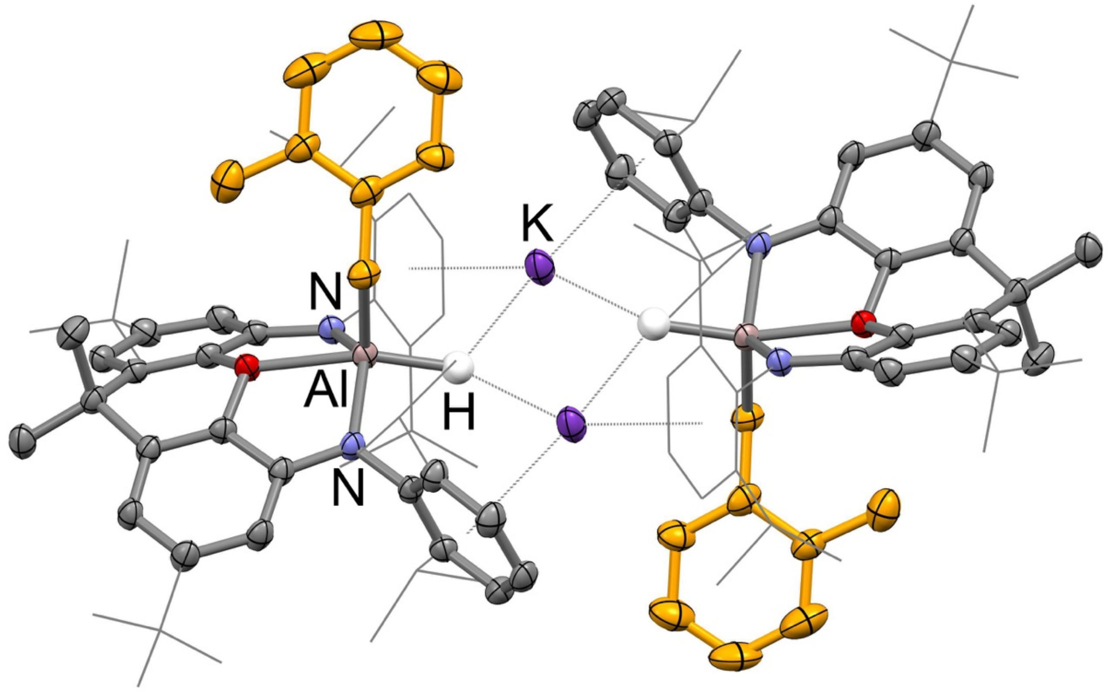

Figure S8. *Molecular structure of **5** as determined by X-ray crystallography. o-Xylene solvate molecules and most hydrogen atoms omitted for clarity. Parts of the ligand are also shown in wireframe for clarity. Thermal ellipsoids set at the 50% probability level. Key bond lengths (Å) and angles (^{o}): Al-N 1.9462(14), 1.9579(14), Al-O 2.1264(12), Al-C 2.080(3), N-Al-N 130.63(6).*

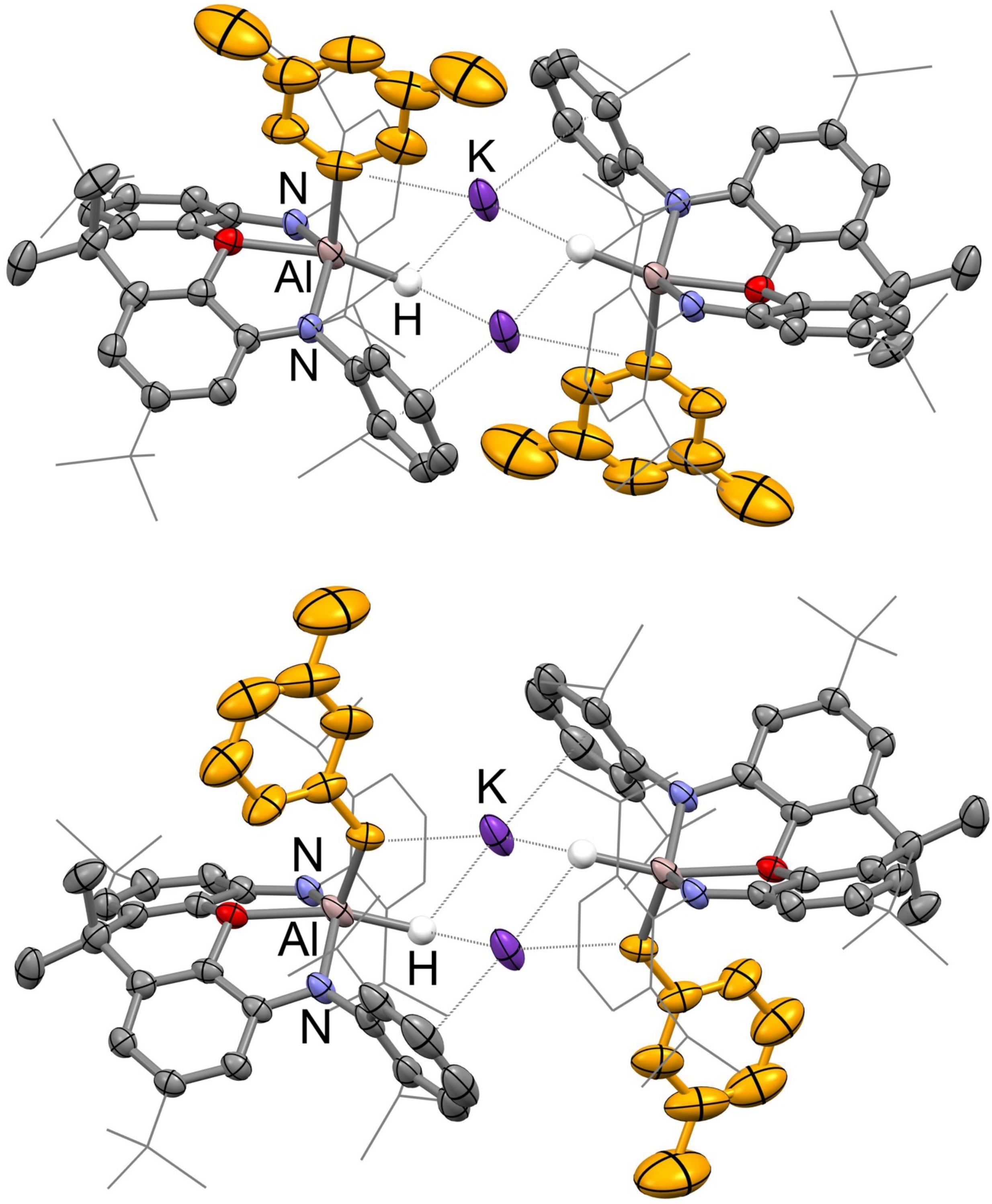

Figure S9. *Molecular structures of **6a** (top) and **6b** (bottom) as determined by X-ray crystallography. Hexane solvate molecules and most hydrogen atoms omitted for clarity. Parts of the ligand are also shown in wireframe for clarity. Thermal ellipsoids set at the 50% probability level. Key bond lengths (Å) and angles (^{o}): Al-N 1.935(2), 1.928(2), Al-O 2.153(2), Al-C 1.960(5), 2.259(9), N-Al-N 130.18(10).*

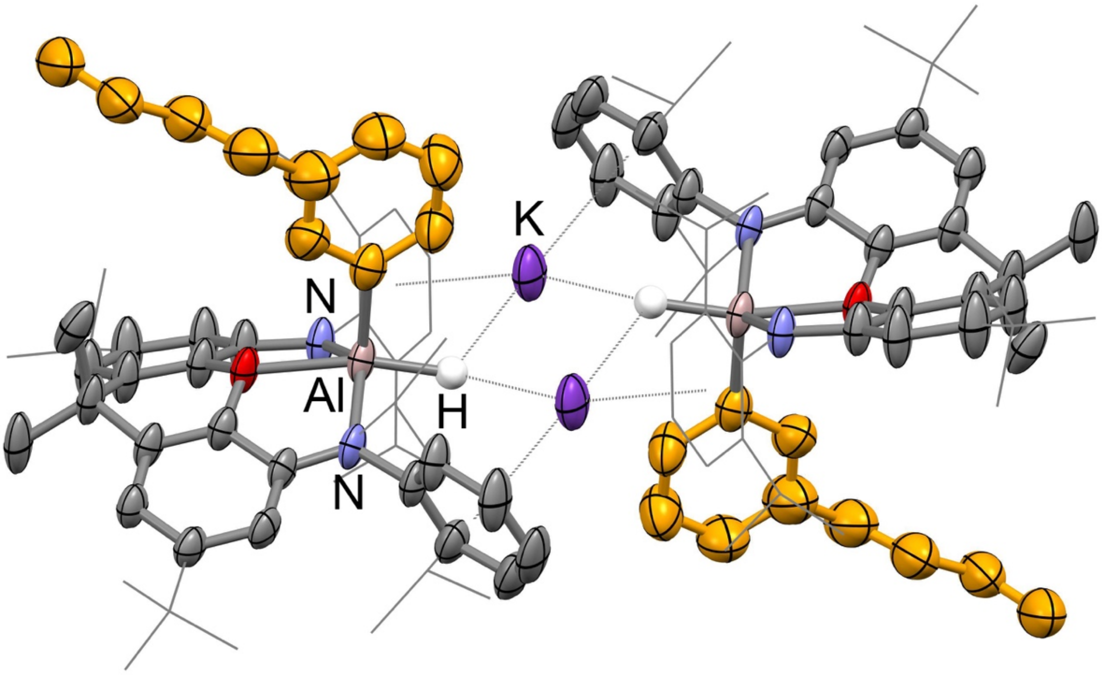

Figure S10. *Molecular structure of **8** as determined by X-ray crystallography. n-Butylbenzene solvate molecules and most hydrogen atoms omitted for clarity. Parts of the ligand are also shown in wireframe for clarity. Thermal ellipsoids set at the 50% probability level. Key bond lengths (Å) and angles (°): Al-N 1.934(2), 1.940(3), Al-O 2.127(2), Al-C 2.005(4), N-Al-N 129.60(12).*

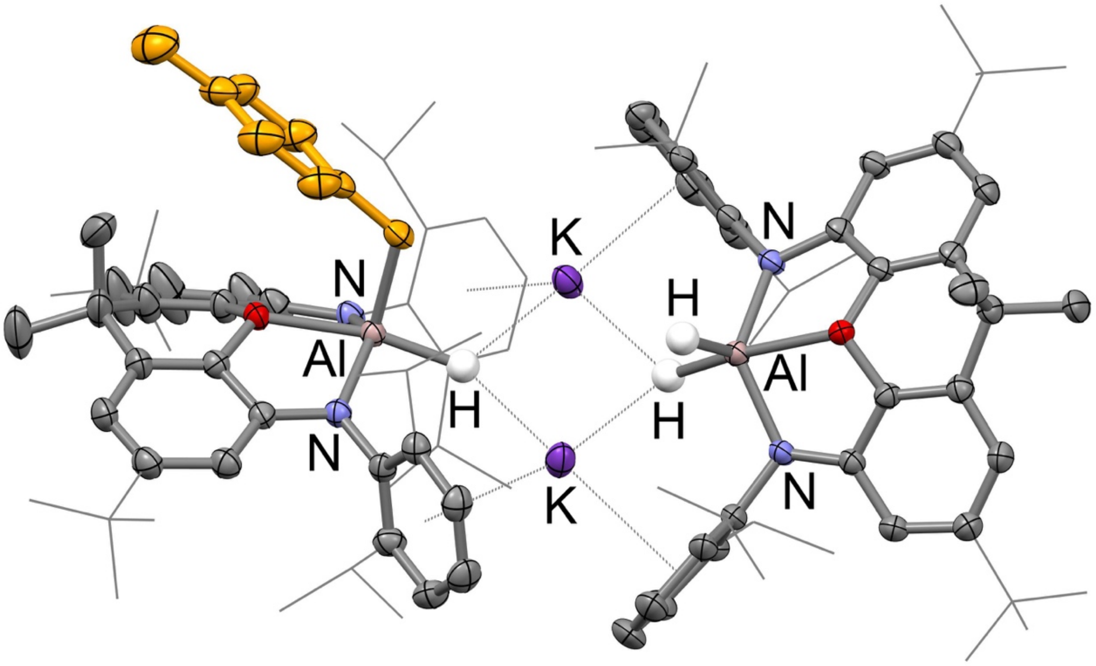

Figure S11. *Molecular structure of K_2[(**NON**)AlH{$CH_2(C_6H_4Me$-4)][(**NON**)AlH_2] as determined by X-ray crystallography. Hexane solvate molecules and most hydrogen atoms omitted for clarity. Parts of the ligand are also shown in wireframe for clarity. Thermal ellipsoids set at the 50% probability level. Key bond lengths (Å) and angles (°): Al-N 1.946(2), 1.933(2), 1.933(2), 1.9175(19), Al-O 2.0811(17), 2.1391(16), Al-C 2.029(2), N-Al-N 128.86(9), 131.82(9).*

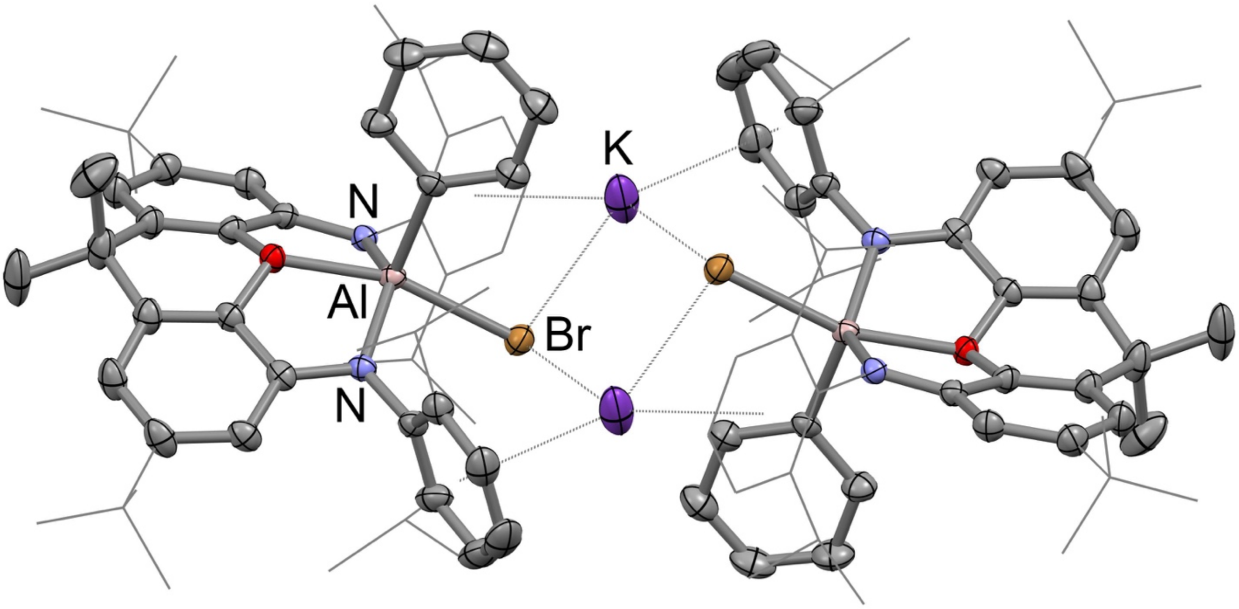

Figure S12. *Molecular structure of K_2[(**NON**)AlBr(Ph)]$_2$ as determined by X-ray crystallography. Benzene solvate molecules and hydrogen atoms omitted for clarity. Parts of the ligand are also shown in wireframe for clarity. Thermal ellipsoids set at the 50% probability level. Key bond lengths (Å) and angles (^{o}): Al-Br 2.4533(6), Al-N 1.9420(16), 1.9324(16), Al-O 2.0256(14), Al-C 2.0088(19), N-Al-N 135.69(7).*

Computational details

The geometry optimizations were performed with the Gaussian16 (Revision C.01) programme[S6] using the PBE1PBE hybrid exchange functional[S7] and Def-TZVP basis set.[S8] In addition, Grimme's empirical dispersion correction with Becke-Johnson damping (GD3BJ)[S9] was used as well as an ultrafine integration grid. The calculations were performed to a simplified monomeric aluminyl anion where the NON-ligand backbone ^{t}Bu groups were replaced by Me groups to reduce computational cost. Full analytical frequency calculations were performed for the optimized structures to ensure the nature of the stationary points found (minima, no imaginary frequencies or a transition state with one imaginary frequency). The energies are Gibbs free energies (kJ mol^{-1}) in the gas phase. The NBO analysis was performed using the NBO 7.0 programme.[S10]

Xyz-coordinates for the optimized structures

Al anion 1'

95

C	-3.69954	-1.49511	1.31014
C	-2.82828	-1.33636	0.21593
C	-2.97910	-2.15168	-0.92355
C	-4.00697	-3.08820	-0.95522
C	-4.87826	-3.23388	0.11282
C	-4.71612	-2.44270	1.23591
N	-1.78805	-0.38881	0.26684
Al	0.01105	-0.83803	1.24103
C	-2.05156	-1.97757	-2.10561
C	-1.83397	-3.26311	-2.89199
C	-3.55129	-0.65959	2.56162
C	-3.22611	-1.53025	3.77180
N	1.76092	-0.51715	0.16289
C	2.09910	0.73264	-0.26989
C	3.25941	1.13640	-0.94984
C	3.42118	2.45269	-1.38110
C	2.42849	3.40337	-1.15358
C	1.26960	3.05290	-0.45333
C	1.17522	1.75311	-0.02227
O	0.05942	1.33438	0.67382
C	-1.11912	1.85843	0.17791
C	-1.17733	3.16893	-0.22279
C	0.10910	3.98382	-0.10218
C	-2.40308	3.62671	-0.71893
C	-3.48884	2.75724	-0.79902
C	-3.35559	1.41466	-0.44705
C	-2.12694	0.90186	0.00553
C	4.68914	2.84324	-2.08713
C	0.27270	4.43327	1.36088
C	0.08195	5.21292	-0.99916
C	-4.82278	3.26291	-1.27167
C	2.81560	-1.44356	0.27576
C	3.80601	-1.27235	1.26432
C	4.84598	-2.19195	1.35198
C	4.91098	-3.28444	0.50625
C	3.91078	-3.47575	-0.43361
C	2.85805	-2.57571	-0.56188
C	3.73984	-0.14077	2.26674
C	4.98436	0.74120	2.22320
C	1.77725	-2.78170	-1.60000
C	1.95441	-1.82431	-2.77744
C	-2.53880	-0.86840	-3.03795
C	-4.78500	0.20509	2.81002
C	1.67535	-4.21801	-2.09353
C	3.48581	-0.68357	3.67132
H	-4.19266	0.73701	-0.57719
H	-2.51577	4.65262	-1.04857
H	2.56276	4.41246	-1.52487
H	4.02746	0.39984	-1.16121
H	1.20796	4.98707	1.48257
H	0.29313	3.57199	2.03036
H	-0.56250	5.07639	1.65249
H	-0.03321	4.93690	-2.04937
H	1.00506	5.78618	-0.88876
H	-0.74416	5.87049	-0.71989
H	-4.12903	-3.71934	-1.82844
H	-5.67424	-3.97057	0.07132
H	-5.38874	-2.56548	2.07968
H	-2.69859	0.00369	2.40498
H	-4.02784	-2.24734	3.97680
H	-3.09241	-0.90892	4.66301
H	-2.29842	-2.08053	3.59959
H	-4.98639	0.85155	1.95296
H	-4.63488	0.84113	3.68769
H	-5.67301	-0.40945	2.99114
H	-1.08988	-1.65185	-1.69993
H	-1.52971	-4.08570	-2.24095
H	-1.05388	-3.11306	-3.64291
H	-2.73871	-3.56892	-3.42671
H	-3.51881	-1.12361	-3.45545
H	-1.83848	-0.73299	-3.86827
H	-2.63009	0.08198	-2.51182
H	3.95112	-4.34716	-1.07645
H	5.72603	-3.99622	0.58940
H	5.60959	-2.05677	2.11228
H	2.88320	0.48093	2.00580
H	5.88269	0.18188	2.50357
H	4.88285	1.57835	2.92041
H	5.13396	1.15039	1.22166
H	2.55623	-1.25734	3.69094
H	3.39691	0.13865	4.38869
H	4.30264	-1.33393	4.00057
H	0.82961	-2.52814	-1.11266
H	1.96379	-0.78685	-2.44378
H	1.13930	-1.94418	-3.49802
H	2.89815	-2.02763	-3.29429
H	2.56129	-4.51252	-2.66518
H	0.81399	-4.32436	-2.75514
H	1.55567	-4.91969	-1.26426
H	4.99509	2.07854	-2.80596
H	4.56849	3.78655	-2.62483
H	5.51414	2.96934	-1.37754
H	-4.71852	4.20301	-1.81852
H	-5.31148	2.53874	-1.92882
H	-5.49848	3.44352	-0.42864

Benzene

12

C	-0.35130	-1.34335	0.00000
C	0.98777	-0.97587	0.00001
C	1.33904	0.36744	-0.00001
C	0.35125	1.34336	0.00000
C	-0.98773	0.97591	0.00001
C	-1.33903	-0.36749	-0.00001

H	-0.62555	-2.39245	-0.00001
H	1.75910	-1.73803	-0.00000
H	2.38472	0.65449	-0.00003
H	0.62564	2.39243	-0.00000
H	-1.75915	1.73797	0.00001
H	-2.38474	-0.65441	-0.00000

nButyl benzene

24

C	2.64576	1.19898	-0.27607
C	1.35213	1.19579	0.22520
C	0.68591	-0.00001	0.48285
C	1.35214	-1.19579	0.22518
C	2.64576	-1.19897	-0.27608
C	3.29752	0.00001	-0.52927
H	3.14854	2.14105	-0.46583
H	0.84929	2.13733	0.42505
H	0.84931	-2.13735	0.42502
H	3.14855	-2.14103	-0.46586
H	4.30957	0.00002	-0.91811
C	-0.73252	-0.00002	0.97376
H	-0.90325	0.87818	1.60546
H	-0.90325	-0.87824	1.60543
C	-1.74689	0.00000	-0.16852
H	-1.57033	0.87540	-0.80435
H	-1.57033	-0.87538	-0.80437
C	-3.18857	-0.00001	0.31432
H	-3.35496	-0.87574	0.95257
H	-3.35496	0.87571	0.95260
C	-4.19318	0.00001	-0.82622
H	-5.22146	0.00001	-0.45690
H	-4.06750	0.88194	-1.46081
H	-4.06750	-0.88190	-1.46084

nButyl benzene activation TS, ortho C-H

119

C	1.72094	-2.37534	-2.02006
C	0.40172	-2.22911	-1.36344
C	-0.53569	-3.29113	-1.75414
C	-0.04187	-4.53838	-2.04538
C	1.33501	-4.77388	-2.19835
C	2.16607	-3.64452	-2.30837
Al	-0.01598	-0.63173	-0.41227
N	-1.84236	-0.26178	-0.03587
C	-2.85928	-1.24501	0.03166
C	-2.88273	-2.17084	1.09041
C	-3.89643	-3.12400	1.13069
C	-4.87008	-3.18609	0.15247
C	-4.81077	-2.30509	-0.91411
C	-3.80835	-1.34818	-1.01024
C	-1.81895	-2.20734	2.16330
C	-1.01087	-3.50233	2.08917
C	-3.72280	-0.52512	-2.28150
C	-3.37608	-1.41226	-3.47695
O	-0.05514	1.30650	-1.07910
C	1.03004	2.04702	-0.59868
C	1.04637	3.40611	-0.79379
C	-0.17570	4.00536	-1.49016
C	-1.38958	3.27295	-0.90717
C	-1.26725	1.91582	-0.74097
C	2.09254	4.11085	-0.19608
C	3.01263	3.44519	0.61568
C	2.85242	2.09263	0.90816
C	1.80047	1.36155	0.34343
C	-2.21022	1.05590	-0.17527
C	-3.41097	1.66494	0.20638
C	-3.60655	3.03344	0.03232
C	-2.60198	3.83789	-0.50951
N	1.36423	0.10758	0.68273
C	1.61911	-0.34896	2.00348
C	2.40205	-1.49951	2.21364
C	2.55746	-1.97360	3.51236
C	1.96487	-1.33406	4.58943
C	1.21224	-0.19379	4.37544
C	1.02414	0.31858	3.09417
C	4.16408	4.19834	1.21607
C	-0.10155	3.72284	-3.00044
C	-0.26805	5.50687	-1.26814
C	-4.92353	3.64110	0.42301
C	3.09942	-2.17828	1.05965
C	4.43085	-1.48923	0.76328
C	0.18203	1.57011	2.93187
C	0.80098	2.75218	3.67549
C	-1.26566	1.36228	3.36402
C	3.29013	-3.67545	1.24657
C	-4.99891	0.27063	-2.54288
C	-2.40439	-2.02047	3.56094
H	0.56439	-2.39097	-0.08817
H	3.49267	1.61321	1.64053
H	2.17715	5.18295	-0.32617
H	-2.76979	4.90376	-0.60750
H	-4.18776	1.05552	0.65520
H	-0.99775	4.10332	-3.49745
H	-0.02497	2.65356	-3.19963
H	0.77565	4.21444	-3.42916
H	-0.32632	5.75274	-0.20588
H	-1.15173	5.90952	-1.76787
H	0.60442	6.00779	-1.69322
H	3.14734	-2.86733	3.67931
H	2.09034	-1.72582	5.59371
H	0.74537	0.30697	5.21801
H	0.15699	1.83275	1.87586
H	-1.74440	0.59152	2.76123
H	-1.83275	2.28789	3.22982
H	-1.33475	1.06743	4.41513
H	0.23068	3.66426	3.47711
H	1.83068	2.91983	3.35540
H	0.80121	2.58334	4.75668

H	2.47431	-2.06224	0.17469
H	4.04084	-3.90951	2.00983
H	3.61335	-4.11579	0.30104
H	2.34889	-4.15853	1.51721
H	4.28628	-0.43156	0.53125
H	4.91232	-1.95875	-0.09918
H	5.10971	-1.56242	1.61990
H	-3.91074	-3.83843	1.94715
H	-5.65413	-3.93417	0.20361
H	-5.54025	-2.38492	-1.71383
H	-2.90888	0.18999	-2.17391
H	-5.85331	-0.39175	-2.71104
H	-4.88182	0.89336	-3.43458
H	-5.23529	0.92472	-1.70116
H	-2.43602	-1.94507	-3.31661
H	-3.27701	-0.80295	-4.38085
H	-4.15835	-2.15578	-3.65728
H	-1.13061	-1.37763	1.98354
H	-3.01436	-1.11677	3.62480
H	-1.59884	-1.94317	4.29624
H	-3.03495	-2.87072	3.83817
H	-1.63199	-4.36147	2.36318
H	-0.16533	-3.45502	2.78047
H	-0.62160	-3.67366	1.08296
H	-0.74380	-5.35283	-2.22048
H	1.70564	-5.75742	-2.46473
H	3.16776	-3.77951	-2.71471
H	-5.30212	3.20468	1.35034
H	-4.83644	4.72048	0.56441
H	-5.67954	3.46795	-0.35035
H	3.95345	5.26881	1.26934
H	4.38744	3.84202	2.22468
H	5.07116	4.06879	0.61681
H	-1.60721	-3.13844	-1.68094
C	2.49246	-1.12524	-2.34051
H	1.81981	-0.43886	-2.88319
H	2.76251	-0.57924	-1.42286
C	3.76778	-1.28907	-3.14904
H	4.45807	-1.95151	-2.61250
H	3.54258	-1.78943	-4.09850
C	4.46331	0.03719	-3.41560
H	3.77908	0.69937	-3.95966
H	4.66360	0.53447	-2.45903
C	5.75946	-0.11203	-4.19624
H	5.58055	-0.58379	-5.16753
H	6.24074	0.85367	-4.37764
H	6.47131	-0.74238	-3.65413

nButyl benzene activation TS, meta C-H

119

Al	-0.33572	0.30247	-0.11912
O	0.85409	-0.52074	-1.56914
N	1.17198	1.43527	0.11067
N	-0.29928	-1.50094	0.49952
C	2.14931	0.00216	-1.48959
C	2.29834	1.04711	-0.57623
C	3.59957	1.54754	-0.45692
H	3.78634	2.35864	0.23838
C	4.64583	1.00643	-1.20070
C	4.42712	-0.06328	-2.07115
H	5.26350	-0.48418	-2.61622
C	3.14505	-0.59221	-2.22437
C	2.73436	-1.77524	-3.10789
C	1.77663	-2.61349	-2.26071
C	1.83602	-3.99354	-2.06018
H	2.51294	-4.60456	-2.64484
C	1.05921	-4.59164	-1.06626
C	0.28138	-3.81544	-0.21064
H	-0.21473	-4.27085	0.63937
C	0.21519	-2.42584	-0.37077
C	0.87325	-1.91857	-1.49426
C	1.99889	-1.23842	-4.34759
H	2.66558	-0.59714	-4.92999
H	1.67112	-2.07027	-4.97650
H	1.12112	-0.65615	-4.06608
C	3.94128	-2.58421	-3.55659
H	4.49734	-2.98151	-2.70504
H	3.62604	-3.41935	-4.18581
H	4.61324	-1.96226	-4.15188
C	1.11592	2.75114	0.63151
C	1.28753	3.85753	-0.23094
C	1.24833	5.13896	0.30401
H	1.38028	5.98930	-0.35749
C	1.00016	5.35458	1.64897
H	0.96188	6.36373	2.04531
C	0.76680	4.26966	2.47181
H	0.53188	4.43187	3.51858
C	0.81449	2.96499	1.98879
C	0.49627	1.83496	2.94088
H	0.59844	0.89408	2.39412
C	-0.94494	1.92517	3.44030
H	-1.07921	2.80386	4.07956
H	-1.65069	1.99827	2.60981
H	-1.19441	1.03488	4.02361
C	1.46854	1.78646	4.11728
H	1.27857	0.89630	4.72314
H	2.50702	1.75643	3.78051
H	1.34971	2.66480	4.75894
C	1.42181	3.71460	-1.73526
H	1.45890	2.65374	-1.97775
C	0.19778	4.28862	-2.44831
H	0.09547	5.36112	-2.25736
H	0.29332	4.14805	-3.52957
H	-0.72094	3.79835	-2.11897
C	2.70583	4.35050	-2.26234
H	3.58584	3.92631	-1.77492
H	2.79905	4.18101	-3.33901
H	2.71078	5.43172	-2.09503

C	-0.33362	-1.83847	1.87952
C	0.87533	-2.04965	2.57403
C	0.82538	-2.28638	3.94565
H	1.75299	-2.43495	4.48977
C	-0.37843	-2.31624	4.62596
H	-0.39650	-2.48968	5.69716
C	-1.56126	-2.12183	3.93098
H	-2.50524	-2.14462	4.46308
C	-1.56426	-1.88452	2.55987
C	-2.86141	-1.72383	1.80775
H	-2.68804	-1.05391	0.96395
C	-3.30302	-3.06131	1.21465
H	-3.49377	-3.79903	2.00198
H	-4.22189	-2.92663	0.63700
H	-2.54151	-3.46494	0.54361
C	-3.97814	-1.09726	2.62826
H	-3.64319	-0.17454	3.10653
H	-4.80380	-0.83168	1.96504
H	-4.36251	-1.77562	3.39866
C	2.23134	-2.02148	1.89440
H	2.08024	-1.84087	0.83171
C	2.94378	-3.36622	2.02576
H	3.18666	-3.58499	3.07006
H	2.32253	-4.17721	1.64217
H	3.87839	-3.35608	1.45753
C	3.11799	-0.89149	2.40693
H	4.07954	-0.90398	1.88579
H	2.65586	0.07788	2.22429
H	3.30829	-0.98339	3.48018
C	-3.19233	0.31134	-0.79070
H	-3.02783	-0.60759	-1.35016
C	-2.05051	1.09827	-0.28151
C	-2.36981	2.53688	-0.21112
H	-1.58254	3.28286	-0.21984
C	-3.66272	2.90959	0.04218
H	-3.88023	3.96385	0.21119
C	-4.72956	1.98838	0.02277
H	-5.74884	2.31835	0.20081
C	-4.48132	0.72049	-0.53622
C	-5.64070	-0.13006	-0.98521
H	-6.39989	-0.15858	-0.19160
H	-5.30292	-1.16309	-1.13328
C	-6.29617	0.36760	-2.27042
H	-5.53574	0.40174	-3.05925
H	-6.62525	1.40210	-2.12264
C	-7.46936	-0.48762	-2.72248
H	-7.13019	-1.52175	-2.86138
H	-8.21966	-0.52178	-1.92256
C	-8.11501	0.01243	-4.00552
H	-8.95588	-0.61632	-4.31388
H	-7.39112	0.02816	-4.82597
H	-8.48858	1.03361	-3.88234
H	-1.85657	0.75296	0.93428
C	6.02102	1.59587	-1.06850
H	6.23981	1.86335	-0.03203
H	6.78735	0.89659	-1.40963
H	6.11497	2.50847	-1.66679
C	1.09300	-6.08147	-0.88494
H	0.24866	-6.55703	-1.39444
H	2.01018	-6.51028	-1.29482
H	1.02953	-6.35318	0.17158

nButyl benzene activation TS, para C-H

119

C	-2.37931	-0.47633	-2.38633
C	-1.80523	0.44039	-1.37987
C	-2.56058	1.70563	-1.32638
C	-3.90809	1.68224	-1.57167
C	-4.56881	0.55149	-2.09951
C	-3.73548	-0.45516	-2.61474
Al	-0.11509	0.10104	-0.58631
N	0.74085	1.52470	0.33693
C	0.07139	2.64814	0.88095
C	-0.77861	2.49911	1.99190
C	-1.42194	3.62194	2.50486
C	-1.25141	4.87286	1.94404
C	-0.45257	5.00393	0.82085
C	0.19585	3.91139	0.25902
C	-1.06056	1.15802	2.62958
C	-2.53163	0.77426	2.47550
C	0.93177	4.11475	-1.05214
C	-0.04142	4.48248	-2.17194
O	1.73119	-0.06744	-1.46162
C	2.17406	-1.39552	-1.48994
C	3.47213	-1.65634	-1.85539
C	4.34940	-0.44128	-2.15735
C	3.98964	0.60412	-1.09616
C	2.65604	0.75263	-0.80568
C	3.89757	-2.98152	-1.75033
C	3.04502	-3.95166	-1.22038
C	1.79555	-3.59904	-0.71644
C	1.34548	-2.27483	-0.78802
C	2.10316	1.59293	0.16200
C	3.02571	2.38266	0.85704
C	4.38916	2.29898	0.58416
C	4.87340	1.40978	-0.37738
N	0.26923	-1.71509	-0.15139
C	-0.17576	-2.31992	1.05511
C	-1.48582	-2.82556	1.14319
C	-1.92921	-3.32206	2.36502
C	-1.10679	-3.33066	3.48031
C	0.18526	-2.84810	3.37933
C	0.67476	-2.33748	2.17934
C	3.49162	-5.38319	-1.15052
C	4.00226	0.11984	-3.54679
C	5.82826	-0.79317	-2.11818
C	5.34213	3.18959	1.32891
C	-2.37185	-2.86860	-0.07638

C	-2.06734	-4.11021	-0.91366
C	2.09804	-1.81376	2.14310
C	3.10215	-2.91808	2.46751
C	2.30209	-0.61468	3.06300
C	-3.85727	-2.77485	0.23332
C	2.04204	5.15610	-0.93488
C	-0.65000	1.12549	4.09995
H	-1.97332	-0.08733	-0.22631
H	1.19399	-4.33087	-0.18865
H	4.90366	-3.25952	-2.03995
H	5.94086	1.34383	-0.55036
H	2.66312	3.05567	1.62636
H	4.59126	1.01906	-3.74523
H	2.94547	0.37877	-3.61678
H	4.22455	-0.62435	-4.31602
H	6.12027	-1.19255	-1.14483
H	6.43321	0.09171	-2.32736
H	6.06184	-1.53690	-2.88308
H	-2.94194	-3.69976	2.44384
H	-1.47491	-3.71261	4.42711
H	0.82894	-2.85098	4.25356
H	2.31319	-1.47094	1.13262
H	1.65630	0.21267	2.77128
H	3.33689	-0.26603	3.00012
H	2.08743	-0.86554	4.10583
H	4.12423	-2.54584	2.35233
H	2.97461	-3.77132	1.79932
H	2.98539	-3.26893	3.49738
H	-2.13549	-2.00725	-0.70413
H	-4.24426	-3.68123	0.71284
H	-4.40474	-2.62267	-0.69908
H	-4.07223	-1.91736	0.87460
H	-1.01882	-4.13280	-1.21820
H	-2.67957	-4.10584	-1.81994
H	-2.28308	-5.02697	-0.35377
H	-2.07877	3.50328	3.36027
H	-1.75899	5.73638	2.36070
H	-0.35708	5.97441	0.34426
H	1.39644	3.17176	-1.33564
H	1.63560	6.14450	-0.70066
H	2.58780	5.23713	-1.87946
H	2.75521	4.88712	-0.15335
H	-0.80592	3.71379	-2.30326
H	0.49909	4.59384	-3.11717
H	-0.54746	5.42940	-1.96136
H	-0.46557	0.40390	2.10785
H	0.38993	1.43054	4.23467
H	-0.76499	0.11373	4.49826
H	-1.27598	1.79720	4.69519
H	-3.16915	1.43867	3.06794
H	-2.68849	-0.25055	2.82218
H	-2.85296	0.83617	1.43342
H	-2.09179	2.62193	-0.98443
H	-4.48527	2.59659	-1.42377
H	-4.17129	-1.19321	-3.29200
H	-1.74667	-1.16593	-2.93857
H	5.05534	3.28725	2.37861
H	6.36221	2.80177	1.28842
H	5.35316	4.19716	0.89961
H	4.58110	-5.46003	-1.16551
H	3.12596	-5.86803	-0.24208
H	3.10767	-5.95498	-2.00158
C	-6.05404	0.52059	-2.27623
H	-6.31154	-0.08174	-3.16056
H	-6.43220	1.53505	-2.47674
C	-6.82421	-0.04092	-1.07883
H	-6.46551	-1.05689	-0.87528
H	-6.56905	0.54921	-0.19031
C	-8.33298	-0.05753	-1.27098
H	-8.68085	0.96249	-1.47769
H	-8.57495	-0.64427	-2.16625
C	-9.08504	-0.62023	-0.07484
H	-8.88162	-0.03249	0.82553
H	-10.16801	-0.62396	-0.23380
H	-8.77426	-1.64876	0.13266

TS, benzene activation

107

C	-0.89422	-2.37103	-3.02889
C	-1.49711	-1.58882	-1.92685
C	-2.96999	-1.56315	-2.02700
C	-3.61745	-2.64479	-2.56337
C	-2.92841	-3.68351	-3.22363
C	-1.58557	-3.43827	-3.55171
Al	-0.41945	-0.48631	-0.82039
N	-1.27014	0.83170	0.25345
C	-2.62436	0.78294	0.66549
C	-3.04793	-0.17906	1.60032
C	-4.38463	-0.20005	1.98840
C	-5.30216	0.69388	1.47117
C	-4.88890	1.60524	0.51427
C	-3.57048	1.65416	0.07942
C	-2.11886	-1.22614	2.17024
C	-2.54003	-2.62907	1.73511
C	-3.23194	2.58024	-1.07352
C	-3.94096	2.13710	-2.35307
O	0.77677	1.10051	-1.32458
C	2.13517	0.76678	-1.26618
C	3.07095	1.76594	-1.37716
C	2.53034	3.19002	-1.50832
C	1.34806	3.27677	-0.53705
C	0.49720	2.19974	-0.50556
C	4.40449	1.39769	-1.19187
C	4.72652	0.08717	-0.83424
C	3.72413	-0.84503	-0.57785
C	2.37508	-0.50267	-0.73371
C	-0.60109	2.03014	0.33900
C	-0.86088	3.10639	1.19481

C	-0.05185	4.24037	1.18541
C	1.05252	4.32500	0.33508
N	1.27446	-1.20060	-0.31199
C	1.43293	-2.08556	0.78829
C	1.16743	-3.45773	0.62776
C	1.23536	-4.28621	1.74372
C	1.56160	-3.78568	2.99392
C	1.83781	-2.43821	3.13864
C	1.78055	-1.56755	2.05292
C	6.16474	-0.31473	-0.68041
C	2.01726	3.42249	-2.93963
C	3.59578	4.22655	-1.18753
C	-0.38654	5.38759	2.09533
C	0.85955	-4.02444	-0.73537
C	2.15289	-4.33793	-1.48662
C	2.08494	-0.09968	2.28601
C	3.51192	0.09514	2.79481
C	1.08401	0.56177	3.22752
C	-0.06111	-5.23450	-0.70771
C	-3.54891	4.03914	-0.75416
C	-2.02555	-1.14334	3.69207
H	-1.25865	-2.20265	-0.83662
H	3.97579	-1.81983	-0.17478
H	5.19490	2.13263	-1.28412
H	1.68231	5.20594	0.36826
H	-1.69840	3.03892	1.88055
H	1.58325	4.42220	-3.02411
H	1.25413	2.69242	-3.21042
H	2.84290	3.33504	-3.65071
H	3.98582	4.10149	-0.17539
H	3.18356	5.23367	-1.28000
H	4.42648	4.14750	-1.89215
H	1.01798	-5.34182	1.62889
H	1.59890	-4.44644	3.85411
H	2.08936	-2.04430	4.11853
H	2.00941	0.42293	1.33411
H	0.07557	0.51376	2.81833
H	1.33740	1.61719	3.36252
H	1.07782	0.08212	4.21059
H	3.74329	1.16135	2.87345
H	4.23473	-0.35958	2.11546
H	3.64356	-0.35218	3.78474
H	0.34267	-3.25958	-1.31756
H	0.43552	-6.12917	-0.31446
H	-0.40067	-5.44816	-1.72328
H	-0.95282	-5.03486	-0.10984
H	2.77498	-3.44590	-1.58926
H	1.91930	-4.70135	-2.49135
H	2.73606	-5.10603	-0.96648
H	-4.70884	-0.94457	2.70798
H	-6.33895	0.66374	1.78917
H	-5.61602	2.27433	0.06516
H	-2.16238	2.51255	-1.26616
H	-4.62187	4.18736	-0.59965
H	-3.24083	4.68439	-1.58205
H	-3.02629	4.36946	0.14567
H	-3.67588	1.11187	-2.62047
H	-3.66012	2.79213	-3.18380
H	-5.02815	2.18630	-2.23991
H	-1.11775	-1.04240	1.77170
H	-1.76074	-0.13763	4.02541
H	-1.26429	-1.83756	4.05819
H	-2.97932	-1.41028	4.15730
H	-3.49661	-2.90450	2.19114
H	-1.78803	-3.35834	2.04764
H	-2.65023	-2.69458	0.65033
H	-3.54311	-0.76258	-1.57142
H	-4.70658	-2.66812	-2.53513
H	-3.46931	-4.50762	-3.67561
H	-1.10097	-4.06154	-4.30428
H	0.08800	-2.11708	-3.41865
H	-0.73132	5.03332	3.06963
H	0.47910	6.03425	2.25396
H	-1.18696	6.00323	1.67109
H	6.80038	0.55229	-0.48709
H	6.29192	-1.02346	0.14155
H	6.53483	-0.80054	-1.58910

nButyl benzene activation product, ortho

119

C	0.48257	0.70072	3.03542
C	-0.10605	1.41040	1.97164
C	-0.62220	2.67897	2.27103
C	-0.57566	3.23291	3.54493
C	-0.00593	2.50673	4.57897
C	0.51720	1.24820	4.31799
Al	-0.28633	0.94591	0.01330
N	-2.07611	0.11823	-0.27088
C	-3.24618	0.86906	-0.01244
C	-3.75884	1.74488	-0.98909
C	-4.91562	2.46429	-0.70613
C	-5.56891	2.32815	0.50791
C	-5.05604	1.47208	1.46554
C	-3.89546	0.74054	1.23270
C	-3.08133	1.86591	-2.33639
C	-3.29017	3.22280	-2.99434
C	-3.37220	-0.17814	2.31764
C	-3.20691	0.54042	3.65316
O	0.01613	-1.25018	-0.10974
C	1.21132	-1.66665	-0.64130
C	1.45755	-2.98654	-0.91483
C	0.37208	-4.00478	-0.57165
C	-0.99079	-3.33256	-0.73052
C	-1.08522	-1.99422	-0.45162
C	2.71047	-3.28515	-1.46716
C	3.62413	-2.26852	-1.73014
C	3.29986	-0.93325	-1.48348
C	2.05136	-0.58790	-0.94945

C	-2.24529	-1.21276	-0.50686
C	-3.41803	-1.90889	-0.82688
C	-3.38426	-3.27644	-1.10532
C	-2.18531	-3.98228	-1.07038
N	1.52584	0.64722	-0.72914
C	2.30312	1.75184	-1.14656
C	3.24946	2.32332	-0.28007
C	4.01486	3.39626	-0.72854
C	3.85393	3.90714	-2.00430
C	2.90809	3.34864	-2.84867
C	2.12296	2.27650	-2.43974
C	4.98074	-2.60075	-2.28440
C	0.53373	-4.40910	0.90448
C	0.48441	-5.24895	-1.44441
C	-4.66767	-3.98600	-1.43316
C	3.45843	1.78638	1.11685
C	4.83412	1.13877	1.26279
C	1.11336	1.66945	-3.38605
C	1.79313	0.76276	-4.41110
C	0.25208	2.72416	-4.06801
C	3.24081	2.86238	2.17550
C	-4.26615	-1.40653	2.48945
C	-3.51425	0.74216	-3.27652
H	-0.57194	2.37001	-0.72246
H	4.00000	-0.14692	-1.74389
H	2.97728	-4.30942	-1.69785
H	-2.18693	-5.04060	-1.30201
H	-4.35745	-1.36861	-0.87400
H	-0.24357	-5.12447	1.18740
H	0.44815	-3.53542	1.55243
H	1.51541	-4.86248	1.06899
H	0.37785	-5.00116	-2.50237
H	-0.28592	-5.97491	-1.17709
H	1.45057	-5.73546	-1.29623
H	4.74822	3.83934	-0.06172
H	4.45868	4.74402	-2.33887
H	2.77563	3.75583	-3.84587
H	0.45203	1.04767	-2.77939
H	-0.22233	3.36824	-3.32553
H	-0.53363	2.24329	-4.65803
H	0.83572	3.35120	-4.74924
H	1.04904	0.29705	-5.06478
H	2.35779	-0.03094	-3.91800
H	2.48453	1.33622	-5.03775
H	2.70220	1.01636	1.27162
H	3.97836	3.66661	2.08699
H	3.32824	2.43354	3.17758
H	2.24270	3.29474	2.08968
H	4.96408	0.32587	0.54511
H	4.95898	0.72637	2.26867
H	5.63314	1.86936	1.10009
H	-5.31555	3.14455	-1.44919
H	-6.47168	2.89641	0.70847
H	-5.55979	1.37416	2.42202
H	-2.38306	-0.51518	2.00268
H	-5.27145	-1.11091	2.80796
H	-3.85366	-2.07134	3.25477
H	-4.35095	-1.97098	1.55996
H	-2.57450	1.42210	3.55330
H	-2.73773	-0.12768	4.38101
H	-4.17330	0.84995	4.06411
H	-2.01116	1.75506	-2.14779
H	-3.29651	-0.23808	-2.85065
H	-2.98734	0.82152	-4.23307
H	-4.58997	0.79582	-3.47666
H	-4.33248	3.38255	-3.28895
H	-2.68487	3.29286	-3.90103
H	-2.99344	4.03484	-2.32609
H	-0.98346	4.22276	3.72746
H	0.03884	2.91690	5.58331
H	0.97372	0.68948	5.12989
H	-5.22847	-3.45508	-2.20761
H	-4.47826	-5.00140	-1.78815
H	-5.31729	-4.05509	-0.55422
H	5.01916	-3.63110	-2.64479
H	5.24237	-1.93971	-3.11526
H	5.75849	-2.48532	-1.52220
H	-1.06709	3.25925	1.46593
C	1.10117	-0.65322	2.79256
H	0.36554	-1.44142	3.00481
H	1.31488	-0.73685	1.72833
C	2.39049	-0.94623	3.54956
H	3.04816	-0.07207	3.48205
H	2.19427	-1.09677	4.61782
C	3.11606	-2.16244	2.99386
H	2.46438	-3.04053	3.06947
H	3.29132	-2.01283	1.92233
C	4.43460	-2.44081	3.69604
H	4.28468	-2.61912	4.76577
H	4.93667	-3.31765	3.27811
H	5.11594	-1.59033	3.59687

nButyl benzene activation product, meta

119

Al	0.51813	-0.64605	-0.80986
O	-0.12183	0.65497	0.87240
N	-0.92691	-1.62950	0.12982
N	2.16201	0.19200	-0.09556
C	-1.06712	0.10706	1.70513
C	-1.51882	-1.13889	1.25899
C	-2.49422	-1.73971	2.06679
H	-2.89783	-2.70428	1.77850
C	-2.92679	-1.12804	3.24424
C	-2.39451	0.09240	3.65249
H	-2.73237	0.53518	4.58187
C	-1.43320	0.73822	2.86632
C	-0.79031	2.09334	3.15965
C	0.65091	2.04562	2.65418

C	1.75458	2.67595	3.24159
H	1.63622	3.26447	4.14350
C	3.01856	2.54590	2.67164
C	3.21503	1.75924	1.53551
H	4.21472	1.62624	1.13643
C	2.14237	1.08642	0.93281
C	0.89133	1.32885	1.51062
C	-1.55197	3.17081	2.36560
H	-2.59953	3.20193	2.67858
H	-1.10020	4.15291	2.53087
H	-1.52190	2.95798	1.29566
C	-0.85228	2.43340	4.64247
H	-0.32343	1.69013	5.24252
H	-0.40639	3.41263	4.82893
H	-1.88954	2.48028	4.98074
C	-1.58738	-2.71939	-0.48597
C	-2.81671	-2.51098	-1.14307
C	-3.48101	-3.60222	-1.69541
H	-4.43091	-3.44378	-2.19669
C	-2.94661	-4.87557	-1.63043
H	-3.47490	-5.71558	-2.07015
C	-1.72068	-5.06880	-1.01419
H	-1.29657	-6.06544	-0.98357
C	-1.02723	-4.01088	-0.43542
C	0.29712	-4.23666	0.25935
H	0.92425	-3.38134	-0.00638
C	1.01637	-5.49389	-0.20930
H	0.48438	-6.40382	0.08701
H	1.13444	-5.50354	-1.29560
H	2.01089	-5.54188	0.23837
C	0.13711	-4.25071	1.77852
H	1.11308	-4.35396	2.26403
H	-0.32516	-3.33174	2.13911
H	-0.48849	-5.09316	2.09249
C	-3.42905	-1.13320	-1.28476
H	-2.71434	-0.41505	-0.88191
C	-3.65070	-0.77282	-2.75080
H	-4.39796	-1.42452	-3.21481
H	-4.00316	0.25840	-2.83573
H	-2.72243	-0.85056	-3.31715
C	-4.73255	-1.00007	-0.49943
H	-4.57824	-1.20709	0.56084
H	-5.12778	0.01628	-0.59369
H	-5.49208	-1.69126	-0.87967
C	3.41139	-0.01053	-0.72508
C	4.19882	-1.12207	-0.37262
C	5.44338	-1.28335	-0.97162
H	6.05768	-2.13687	-0.70688
C	5.91319	-0.37191	-1.90398
H	6.88889	-0.51098	-2.35870
C	5.12404	0.70645	-2.26219
H	5.48363	1.41077	-3.00604
C	3.86834	0.90098	-1.69337
C	3.02332	2.07823	-2.12663
H	2.03960	1.95172	-1.67202
C	3.60568	3.40317	-1.63763
H	4.59907	3.57600	-2.06551
H	2.96066	4.23469	-1.93749
H	3.69364	3.41728	-0.54976
C	2.82581	2.10308	-3.63933
H	2.43620	1.14939	-3.99712
H	2.10452	2.87802	-3.91254
H	3.76373	2.31255	-4.16419
C	3.70534	-2.10091	0.66802
H	2.61403	-2.06689	0.62862
C	4.12573	-1.67445	2.07412
H	5.21732	-1.67364	2.16455
H	3.76484	-0.67239	2.30928
H	3.72226	-2.36655	2.81999
C	4.13634	-3.53453	0.39339
H	3.66603	-4.20694	1.11564
H	3.84236	-3.85001	-0.60998
H	5.21863	-3.66327	0.49278
C	-1.04510	1.72875	-1.81623
H	-1.13261	1.99987	-0.76712
C	-0.32205	0.58450	-2.15747
C	-0.24219	0.29713	-3.52678
H	0.30490	-0.58597	-3.84931
C	-0.84455	1.10407	-4.48359
H	-0.75935	0.85643	-5.53812
C	-1.57082	2.22401	-4.09627
H	-2.05629	2.84786	-4.84251
C	-1.68217	2.54603	-2.74814
C	-2.50938	3.70569	-2.26739
H	-2.78975	4.34557	-3.11185
H	-1.90957	4.32563	-1.58942
C	-3.76541	3.25006	-1.52792
H	-3.47723	2.53554	-0.74902
H	-4.40780	2.69315	-2.22052
C	-4.55173	4.38731	-0.89746
H	-3.89349	4.94079	-0.21697
H	-4.85495	5.09967	-1.67469
C	-5.77562	3.90061	-0.13799
H	-6.32880	4.72747	0.31597
H	-5.48775	3.21120	0.66095
H	-6.46085	3.36349	-0.80067
H	1.20933	-1.87904	-1.61791
C	-3.98979	-1.79915	4.06749
H	-3.77876	-2.86422	4.19627
H	-4.06778	-1.34608	5.05832
H	-4.97082	-1.71980	3.58694
C	4.19397	3.25967	3.27746
H	4.48515	4.12566	2.67353
H	3.96318	3.61890	4.28289
H	5.06611	2.60340	3.34242

nButyl benzene activation product, para

119

C	-1.96265	-1.35858	1.01792
C	-1.47985	-1.16743	-0.28183
C	-2.23317	-1.76025	-1.30194
C	-3.39064	-2.48671	-1.05011
C	-3.85741	-2.65221	0.24976
C	-3.11989	-2.07954	1.28312
Al	0.13554	-0.09722	-0.79742
N	1.77659	-1.14435	-0.44950
C	2.13921	-2.21347	-1.30284
C	2.85650	-1.96584	-2.48973
C	3.22349	-3.04197	-3.29120
C	2.90113	-4.34364	-2.94215
C	2.18019	-4.57854	-1.78615
C	1.78034	-3.53252	-0.95966
C	3.23445	-0.55076	-2.86699
C	3.49899	-0.37453	-4.35576
C	0.96360	-3.84630	0.27620
C	-0.31352	-4.60611	-0.07132
O	0.69701	0.32261	1.32099
C	0.52334	1.62987	1.71460
C	0.99176	2.06978	2.92590
C	1.67807	1.04254	3.82412
C	2.45415	0.08324	2.92296
C	1.89026	-0.23805	1.71447
C	0.78573	3.42154	3.22613
C	0.14702	4.25173	2.30853
C	-0.25872	3.76505	1.06539
C	-0.04334	2.42496	0.71220
C	2.44752	-1.06894	0.73736
C	3.65614	-1.68182	1.09569
C	4.25723	-1.41703	2.32660
C	3.67398	-0.53093	3.22872
N	-0.25714	1.81460	-0.48675
C	-0.90227	2.58543	-1.48043
C	-2.29918	2.74653	-1.45207
C	-2.90529	3.54222	-2.42039
C	-2.16065	4.15837	-3.41019
C	-0.78738	3.97500	-3.44681
C	-0.14052	3.19525	-2.49439
C	-0.11552	5.69104	2.65109
C	0.59358	0.23893	4.56404
C	2.58649	1.71053	4.84706
C	5.54378	-2.11031	2.67604
C	-3.14974	2.07485	-0.39677
C	-3.74958	3.08925	0.57522
C	1.36175	3.03057	-2.51522
C	2.04791	4.11023	-1.67895
C	1.94215	2.99297	-3.92144
C	-4.24492	1.21318	-1.01821
C	1.77909	-4.61799	1.31249
C	4.42700	-0.05228	-2.05279
H	0.17940	-0.07089	-2.42608
H	-0.70424	4.43754	0.34043
H	1.12451	3.83295	4.16939

H	4.17318	-0.32367	4.16774
H	4.13828	-2.34979	0.39003
H	1.05471	-0.52912	5.19127
H	-0.07358	-0.25443	3.85515
H	-0.00515	0.90291	5.19384
H	3.37201	2.29252	4.36077
H	3.05465	0.96134	5.48899
H	2.00984	2.37570	5.49327
H	-3.98298	3.67245	-2.39991
H	-2.64887	4.77295	-4.15988
H	-0.20928	4.44799	-4.23293
H	1.57135	2.06812	-2.04301
H	1.46019	2.22199	-4.52643
H	3.01170	2.77221	-3.87407
H	1.83517	3.95337	-4.43530
H	3.13094	3.95207	-1.66508
H	1.68973	4.10119	-0.64869
H	1.85339	5.10303	-2.09884
H	-2.49512	1.40956	0.16750
H	-4.98909	1.82340	-1.54059
H	-4.76086	0.64220	-0.24122
H	-3.82389	0.49895	-1.72631
H	-2.96915	3.66974	1.07061
H	-4.33289	2.57631	1.34603
H	-4.41734	3.78387	0.05445
H	3.77206	-2.86192	-4.20815
H	3.20156	-5.16969	-3.57891
H	1.90833	-5.59544	-1.52023
H	0.66257	-2.89598	0.71814
H	2.09481	-5.58918	0.91699
H	1.17779	-4.80038	2.20839
H	2.66948	-4.06109	1.60901
H	-0.90416	-4.06232	-0.80873
H	-0.93243	-4.72901	0.82188
H	-0.09220	-5.60235	-0.46773
H	2.36948	0.06725	-2.61194
H	4.23264	-0.11363	-0.98194
H	4.64681	0.99171	-2.29918
H	5.32007	-0.64742	-2.27201
H	4.39934	-0.90813	-4.67747
H	3.65231	0.68220	-4.58334
H	2.65676	-0.72977	-4.95451
H	-1.89981	-1.64628	-2.33131
H	-3.94518	-2.93003	-1.87473
H	-3.46121	-2.20093	2.30932
H	-1.41957	-0.92844	1.85556
H	6.24800	-2.08526	1.84003
H	6.02366	-1.64248	3.53859
H	5.37110	-3.16359	2.92191
H	0.47519	6.00632	3.51417
H	0.12850	6.34918	1.81279
H	-1.17115	5.85372	2.89341
C	-5.15197	-3.36164	0.52407
H	-5.10205	-3.86293	1.49779

H	-5.31026	-4.14575	-0.22541
C	-6.34853	-2.41266	0.51277
H	-6.18477	-1.62599	1.25870
H	-6.38756	-1.90242	-0.45668
C	-7.67395	-3.10691	0.78228
H	-7.82705	-3.89391	0.03388
H	-7.62312	-3.61751	1.75165
C	-8.85706	-2.15216	0.76898
H	-8.94488	-1.65138	-0.19961
H	-9.80004	-2.67044	0.96381
H	-8.74054	-1.37379	1.52877

product, benzene

107

C	0.03007	-0.33044	3.21242
C	0.05358	-1.29209	2.19464
C	0.11107	-2.62752	2.61329
C	0.14427	-2.98877	3.95572
C	0.11923	-2.00770	4.93722
C	0.06125	-0.67183	4.55955
Al	-0.01222	-0.92457	0.22072
N	1.76233	-0.31451	-0.40473
C	2.82873	-1.23324	-0.55354
C	2.95649	-1.99231	-1.73342
C	4.03370	-2.86335	-1.86006
C	4.97832	-2.99279	-0.85460
C	4.83708	-2.25931	0.30864
C	3.76888	-1.38482	0.48555
C	1.94529	-1.84020	-2.84786
C	1.88896	-3.04379	-3.77835
C	3.64662	-0.63417	1.79485
C	3.59559	-1.58729	2.98564
O	0.01453	1.28703	0.51067
C	-1.16758	1.92178	0.20450
C	-1.24317	3.29032	0.16577
C	0.02590	4.06941	0.50562
C	1.21493	3.27872	-0.03844
C	1.13938	1.91036	0.02158
C	-2.48315	3.84188	-0.17769
C	-3.55940	3.01004	-0.47589
C	-3.41114	1.62263	-0.48031
C	-2.17739	1.03013	-0.17380
C	2.10078	1.00809	-0.44436
C	3.27579	1.59230	-0.93710
C	3.41953	2.97904	-0.99083
C	2.39508	3.81907	-0.56167
N	-1.81877	-0.28197	-0.25333
C	-2.84727	-1.19483	-0.58123
C	-3.73646	-1.64092	0.41228
C	-4.76777	-2.50524	0.05519
C	-4.91839	-2.93965	-1.24955
C	-4.02195	-2.51737	-2.21857
C	-2.98247	-1.64803	-1.90656
C	-4.90209	3.60277	-0.79799
C	0.15696	4.14607	2.03738
C	-0.01510	5.48189	-0.06098
C	4.70389	3.56133	-1.50982
C	-3.59038	-1.20418	1.85272
C	-4.73264	-0.28633	2.28403
C	-2.02865	-1.16380	-2.97436
C	-2.52953	0.12666	-3.62214
C	-1.73370	-2.21427	-4.03536
C	-3.47353	-2.39946	2.79367
C	4.77300	0.38270	1.97232
C	2.18105	-0.56052	-3.64811
H	-0.07385	-2.37382	-0.52110
H	-4.24302	0.99079	-0.77190
H	-2.61407	4.91653	-0.21991
H	2.52196	4.89252	-0.63615
H	4.07001	0.95086	-1.30366
H	1.07659	4.67029	2.31201
H	0.19065	3.14625	2.47352
H	-0.69847	4.67761	2.46363
H	-0.10789	5.47107	-1.14888
H	0.89283	6.02685	0.20589
H	-0.85928	6.03594	0.35488
H	-5.45917	-2.84863	0.81864
H	-5.72602	-3.61582	-1.51155
H	-4.13395	-2.87317	-3.23679
H	-1.09026	-0.93144	-2.46550
H	-1.37849	-3.14288	-3.58353
H	-0.96073	-1.84627	-4.71511
H	-2.61479	-2.44088	-4.64379
H	-1.80644	0.48954	-4.35942
H	-2.68078	0.91086	-2.87932
H	-3.48214	-0.04576	-4.13458
H	-2.65825	-0.64236	1.92468
H	-4.40710	-2.97021	2.83644
H	-3.23616	-2.06076	3.80586
H	-2.67395	-3.06881	2.47478
H	-4.78477	0.60153	1.65110
H	-4.58621	0.04111	3.31781
H	-5.69547	-0.80549	2.22855
H	4.13875	-3.45451	-2.76202
H	5.81433	-3.67435	-0.97564
H	5.56404	-2.37330	1.10690
H	2.69980	-0.09361	1.77432
H	5.74724	-0.11587	2.01171
H	4.64097	0.93447	2.90802
H	4.78769	1.10304	1.15271
H	2.79347	-2.31628	2.86947
H	3.40366	-1.02920	3.90619
H	4.54078	-2.12594	3.10849
H	0.97133	-1.75801	-2.35805
H	2.16303	0.32031	-3.00623
H	1.40594	-0.44041	-4.41209
H	3.15229	-0.59469	-4.15337
H	2.80796	-3.15174	-4.36373

H	1.06713	-2.92718	-4.48744
H	1.72563	-3.96991	-3.22182
H	0.13003	-3.41016	1.85779
H	0.18964	-4.03712	4.23746
H	0.14497	-2.28026	5.98788
H	0.04101	0.10532	5.31874
H	-0.01543	0.72240	2.94649
H	5.02503	3.06009	-2.42692
H	4.59678	4.62693	-1.72470
H	5.51248	3.44872	-0.77962
H	-4.81988	4.66798	-1.02566
H	-5.35940	3.10514	-1.65744
H	-5.59414	3.49532	0.04406

Toluene

15

C	0.19429	-1.19534	-0.00934
C	0.90916	0.00028	-0.01186
C	0.19396	1.19552	-0.00933
C	-1.19346	1.19820	0.00213
C	-1.89309	-0.00018	0.00872
C	-1.19298	-1.19846	0.00214
H	0.73380	-2.13741	-0.01845
H	0.73314	2.13776	-0.01842
H	-1.73027	2.14055	0.00188
H	-2.97720	-0.00041	0.01475
H	-1.72954	-2.14095	0.00190
C	2.40860	0.00012	0.00971
H	2.78405	-0.01618	1.03794
H	2.81372	-0.87632	-0.50019
H	2.81345	0.89215	-0.47243

Toluene activation TS, ortho

110

C	-0.75131	-2.45566	-2.89968
C	-1.40541	-1.69671	-1.80875
C	-2.87182	-1.79776	-1.86613
C	-3.44327	-2.94617	-2.35434
C	-2.68641	-3.93533	-3.00645
C	-1.37420	-3.58789	-3.37093
Al	-0.39283	-0.49487	-0.73302
N	-1.33077	0.81107	0.28235
C	-2.68482	0.69683	0.68128
C	-3.06318	-0.24940	1.65080
C	-4.40129	-0.33274	2.02560
C	-5.36279	0.48311	1.46110
C	-4.99169	1.37515	0.46918
C	-3.67277	1.48310	0.04681
C	-2.08259	-1.22041	2.26685
C	-2.41259	-2.65768	1.86585
C	-3.37279	2.36859	-1.14759
C	-4.02231	1.80645	-2.41189
O	0.72078	1.14099	-1.27585
C	2.09433	0.88966	-1.18793
C	2.97227	1.93667	-1.32502
C	2.35213	3.32074	-1.51808
C	1.15062	3.37576	-0.56813
C	0.36388	2.25241	-0.50603
C	4.32227	1.65291	-1.11149
C	4.71462	0.37654	-0.70454
C	3.76397	-0.60281	-0.42645
C	2.40015	-0.34409	-0.60824
C	-0.73671	2.05031	0.32853
C	-1.07545	3.14294	1.13484
C	-0.33434	4.32186	1.09154
C	0.77857	4.43833	0.25623
N	1.33436	-1.09132	-0.18020
C	1.52290	-1.92600	0.95402
C	1.34059	-3.31709	0.84452
C	1.44400	-4.09734	1.99194
C	1.72472	-3.53253	3.22560
C	1.91730	-2.16638	3.32144
C	1.82114	-1.34197	2.20316
C	6.17149	0.06379	-0.52162
C	1.85025	3.46710	-2.96474
C	3.35030	4.42932	-1.22284
C	-0.75257	5.48260	1.94833
C	1.08717	-3.95985	-0.49730
C	2.41028	-4.26048	-1.20018
C	2.03419	0.14921	2.38513
C	3.44118	0.44814	2.89920
C	0.98565	0.78334	3.29314
C	0.21031	-5.20097	-0.43021
C	-3.79246	3.81757	-0.91478
C	-2.01930	-1.08946	3.78667
H	-1.08511	-2.24463	-0.68386
H	4.06603	-1.54763	0.01194
H	5.07035	2.42858	-1.22190
H	1.35452	5.35598	0.26319
H	-1.92003	3.05381	1.80935
H	1.35967	4.43526	-3.09505
H	1.13628	2.68264	-3.21719
H	2.69137	3.40107	-3.65981
H	3.73024	4.36567	-0.20117
H	2.88198	5.40638	-1.36002
H	4.19566	4.37252	-1.91201
H	1.29140	-5.16769	1.91538
H	1.79161	-4.15755	4.11031
H	2.13302	-1.72158	4.28801
H	1.93820	0.63181	1.41425
H	-0.01382	0.66175	2.87733
H	1.17545	1.85601	3.39170
H	0.99699	0.33988	4.29302
H	3.60758	1.52851	2.93940
H	4.19756	0.01265	2.24430
H	3.58787	0.04695	3.90667
H	0.55072	-3.24290	-1.11788
H	0.72602	-6.05286	0.02728

H	-0.08947	-5.48340	-1.44146
H	-0.70640	-5.00156	0.12849
H	3.00449	-3.35276	-1.32947
H	2.22025	-4.68101	-2.19164
H	3.00453	-4.98006	-0.62628
H	-4.69086	-1.06477	2.77227
H	-6.40000	0.40542	1.76956
H	-5.75120	1.97882	-0.01744
H	-2.29733	2.36397	-1.31732
H	-4.87542	3.90214	-0.78407
H	-3.51302	4.43592	-1.77280
H	-3.30866	4.23053	-0.02730
H	-3.68612	0.78629	-2.61097
H	-3.76538	2.42857	-3.27506
H	-5.11273	1.79043	-2.32209
H	-1.08815	-0.98875	1.87684
H	-1.82065	-0.06067	4.09474
H	-1.22371	-1.72588	4.18353
H	-2.96240	-1.40077	4.24607
H	-3.35793	-2.97696	2.31687
H	-1.62327	-3.33163	2.20899
H	-2.50401	-2.75935	0.78200
H	-4.52433	-3.06235	-2.28699
H	-3.16105	-4.81696	-3.42241
H	-0.85930	-4.19069	-4.12105
H	-1.09277	5.14768	2.93107
H	0.07027	6.18588	2.09335
H	-1.58053	6.03166	1.48720
H	6.75528	0.97300	-0.36145
H	6.32975	-0.60016	0.33181
H	6.57781	-0.43939	-1.40502
H	-3.49650	-1.03769	-1.40889
C	0.53877	-1.96402	-3.48339
H	0.45181	-0.91094	-3.79126
H	1.39253	-2.00867	-2.79133
H	0.81832	-2.54170	-4.37096

Toluene activation ortho, product

110

C	0.01117	-1.53012	2.95950
C	0.06580	-1.93034	1.60984
C	0.16490	-3.30661	1.37048
C	0.21063	-4.25323	2.38947
C	0.15392	-3.83374	3.70784
C	0.05394	-2.47597	3.98189
Al	-0.00831	-0.84378	-0.09083
N	1.81531	-0.13357	-0.44711
C	2.86798	-1.01205	-0.79444
C	3.04174	-1.41446	-2.13324
C	4.10200	-2.25788	-2.44910
C	4.98379	-2.70131	-1.47675
C	4.79764	-2.31592	-0.16198
C	3.74464	-1.48215	0.20300
C	2.10784	-0.90827	-3.21055
C	1.98529	-1.85974	-4.39286
C	3.57466	-1.10201	1.65828
C	3.51756	-2.32767	2.56467
O	-0.00328	1.25735	0.57005
C	-1.18264	1.94634	0.42730
C	-1.26276	3.29727	0.64156
C	-0.01019	4.02742	1.12128
C	1.21427	3.32238	0.54081
C	1.14489	1.97009	0.32797
C	-2.51828	3.88695	0.43699
C	-3.60101	3.11469	0.02770
C	-3.45477	1.74718	-0.21176
C	-2.21435	1.11975	-0.03779
C	2.15655	1.16260	-0.20818
C	3.36919	1.81527	-0.46565
C	3.50576	3.18536	-0.23701
C	2.43959	3.93673	0.24732
N	-1.86151	-0.16761	-0.29504
C	-2.88383	-1.02738	-0.75569
C	-3.69601	-1.71178	0.16679
C	-4.71248	-2.53380	-0.31036
C	-4.92969	-2.69072	-1.66859
C	-4.11732	-2.02485	-2.57097
C	-3.09085	-1.19269	-2.13643
C	-4.95137	3.74801	-0.15577
C	0.05280	3.92540	2.65583
C	-0.04117	5.49873	0.72312
C	4.82770	3.84351	-0.51485
C	-3.49699	-1.54611	1.65632
C	-4.59495	-0.67709	2.26830
C	-2.24011	-0.45222	-3.14174
C	-3.01040	0.71196	-3.76327
C	-1.68075	-1.37783	-4.21404
C	-3.39582	-2.88349	2.38042
C	4.67415	-0.14765	2.12330
C	2.51287	0.48505	-3.68926
H	-0.05983	-1.94433	-1.29105
H	-4.29781	1.16409	-0.56635
H	-2.65610	4.94977	0.59482
H	2.56773	5.00193	0.39715
H	4.19794	1.24797	-0.87520
H	0.95603	4.41524	3.03060
H	0.07228	2.88142	2.97226
H	-0.82399	4.40274	3.10268
H	-0.08574	5.61411	-0.36160
H	0.84725	6.01476	1.09228
H	-0.90784	5.99658	1.16237
H	-5.34307	-3.06226	0.39765
H	-5.72684	-3.33608	-2.02386
H	-4.28412	-2.15271	-3.63590
H	-1.39472	-0.03721	-2.58947
H	-1.10344	-2.18410	-3.75794
H	-1.02171	-0.82044	-4.88573
H	-2.47372	-1.81984	-4.82528

H	-2.37270	1.26632	-4.45894
H	-3.35786	1.40511	-2.99456
H	-3.88275	0.35039	-4.31801
H	-2.54465	-1.03129	1.78813
H	-4.33722	-3.44085	2.33607
H	-3.15203	-2.72208	3.43375
H	-2.60540	-3.50057	1.95120
H	-4.62996	0.30590	1.79460
H	-4.41487	-0.53379	3.33831
H	-5.57632	-1.14934	2.15166
H	4.24304	-2.57616	-3.47546
H	5.80722	-3.35580	-1.74461
H	5.47735	-2.67576	0.60405
H	2.61614	-0.58759	1.74271
H	5.65717	-0.62553	2.05453
H	4.51221	0.13745	3.16746
H	4.69104	0.76237	1.52163
H	2.73444	-3.01644	2.24707
H	3.29704	-2.02479	3.59183
H	4.47164	-2.86451	2.57319
H	1.12174	-0.83324	-2.74534
H	2.54027	1.19836	-2.86493
H	1.80083	0.85338	-4.43495
H	3.50587	0.46142	-4.15128
H	2.91758	-1.92958	-4.96253
H	1.21494	-1.50198	-5.07949
H	1.70593	-2.86412	-4.06577
H	0.28888	-5.31000	2.15155
H	0.18599	-4.55296	4.52047
H	0.00845	-2.14226	5.01590
H	5.20715	3.56971	-1.50331
H	4.74395	4.93174	-0.47320
H	5.58323	3.53849	0.21688
H	-4.88274	4.83793	-0.13598
H	-5.40175	3.45339	-1.10785
H	-5.64292	3.44275	0.63650
H	0.20451	-3.64807	0.33874
C	-0.09283	-0.07796	3.33346
H	0.77809	0.48311	2.98308
H	-0.97059	0.38956	2.87900
H	-0.16356	0.04842	4.41680

Toluene activation TS, meta

110

Al	-0.56763	0.27948	-0.65869
O	1.20876	-0.45527	-1.36813
N	0.59111	1.46377	0.27075
N	-0.74107	-1.53714	-0.10437
C	2.28189	0.11707	-0.67688
C	1.92618	1.14129	0.20233
C	2.98719	1.69090	0.93057
H	2.78032	2.48951	1.63457
C	4.28644	1.21335	0.77384
C	4.56157	0.15985	-0.10040
H	5.57601	-0.21171	-0.18196
C	3.53509	-0.41618	-0.84968
C	3.65338	-1.59025	-1.82768
C	2.44956	-2.48949	-1.54488
C	2.47292	-3.87102	-1.34689
H	3.37548	-4.43809	-1.53990
C	1.34594	-4.52683	-0.84790
C	0.21759	-3.80630	-0.46415
H	-0.60156	-4.30414	0.04311
C	0.16906	-2.41647	-0.62895
C	1.25763	-1.85268	-1.29965
C	3.57282	-1.04999	-3.26559
H	4.40421	-0.36623	-3.45587
H	3.62587	-1.87701	-3.97846
H	2.63963	-0.51214	-3.43483
C	4.96592	-2.33718	-1.64950
H	5.06741	-2.73484	-0.63769
H	5.02956	-3.16690	-2.35682
H	5.80931	-1.67257	-1.84884
C	0.22923	2.76093	0.70933
C	0.73507	3.89746	0.03884
C	0.38315	5.16042	0.49806
H	0.77111	6.03404	-0.01609
C	-0.48442	5.32718	1.56415
H	-0.75619	6.32222	1.90031
C	-1.02689	4.21061	2.17051
H	-1.73897	4.33311	2.97996
C	-0.69109	2.92336	1.76063
C	-1.36872	1.75418	2.43748
H	-0.96944	0.83459	2.00200
C	-2.87531	1.77030	2.18319
H	-3.34351	2.62356	2.68470
H	-3.09858	1.83949	1.11614
H	-3.32905	0.85364	2.56912
C	-1.07893	1.71194	3.93602
H	-1.49144	0.79752	4.37099
H	-0.00614	1.73442	4.13951
H	-1.53485	2.56557	4.44671
C	1.57424	3.80253	-1.22139
H	1.77762	2.75154	-1.42045
C	0.80602	4.34084	-2.42809
H	0.57016	5.40221	-2.30521
H	1.40935	4.23404	-3.33509
H	-0.13244	3.80166	-2.57525
C	2.91944	4.50898	-1.07297
H	3.48385	4.11050	-0.22762
H	3.52069	4.37386	-1.97679
H	2.78833	5.58429	-0.91949
C	-1.41315	-1.91453	1.08928
C	-0.67303	-2.09844	2.27560
C	-1.35955	-2.37700	3.45498
H	-0.79777	-2.50495	4.37511
C	-2.73916	-2.47382	3.47740
H	-3.25736	-2.67939	4.40859

C	-3.45514	-2.30476	2.30334
H	-4.53640	-2.37954	2.31945
C	-2.81535	-2.02722	1.09911
C	-3.60464	-1.89342	-0.17874
H	-3.06752	-1.21660	-0.84499
C	-3.68306	-3.23783	-0.90110
H	-4.21104	-3.98253	-0.29509
H	-4.21687	-3.11973	-1.84834
H	-2.68536	-3.62291	-1.12432
C	-4.98994	-1.29524	0.01352
H	-4.94046	-0.37146	0.59336
H	-5.40789	-1.03686	-0.96130
H	-5.67946	-1.98784	0.50991
C	0.83980	-1.99628	2.32676
H	1.20536	-1.79081	1.32228
C	1.46828	-3.31463	2.77417
H	1.19348	-3.55384	3.80602
H	1.14628	-4.13833	2.13489
H	2.55902	-3.25049	2.72234
C	1.31776	-0.84706	3.20807
H	2.41080	-0.80706	3.21146
H	0.95351	0.10819	2.83198
H	0.97552	-0.96154	4.24069
C	-2.73984	0.18095	-2.62347
H	-2.26994	-0.70789	-3.03939
C	-2.03575	1.00374	-1.61764
C	-2.42836	2.42334	-1.70271
H	-1.78118	3.20397	-1.31743
C	-3.69613	2.72826	-2.12050
H	-4.02414	3.76664	-2.07910
C	-4.56570	1.76227	-2.66603
H	-5.54954	2.04683	-3.02666
C	-4.00783	0.52355	-3.03464
H	-2.43294	0.62933	-0.46424
C	5.40187	1.85436	1.54911
H	5.08572	2.10577	2.56423
H	6.26969	1.19469	1.61537
H	5.72782	2.78390	1.07020
C	1.36117	-6.01852	-0.67965
H	0.88413	-6.51348	-1.53177
H	2.38240	-6.39900	-0.60587
H	0.81667	-6.32114	0.21816
C	-4.76941	-0.39157	-3.95404
H	-5.69864	-0.73647	-3.48490
H	-5.05096	0.11251	-4.88534
H	-4.17759	-1.27543	-4.20768

Toluene activation product, meta

110

Al	0.01916	0.91128	0.24953
O	-0.01363	-1.31812	0.15517
N	-1.75308	0.41976	-0.48175
N	1.82890	0.35167	-0.31215
C	-1.13668	-1.84664	-0.43773
C	-2.09377	-0.87563	-0.74826
C	-3.26710	-1.36449	-1.33906
H	-4.05792	-0.66800	-1.59610
C	-3.41354	-2.72178	-1.62713
C	-2.39348	-3.62561	-1.34072
H	-2.52254	-4.67071	-1.59583
C	-1.21488	-3.18499	-0.72810
C	-0.03039	-4.05933	-0.31892
C	1.24198	-3.23771	-0.51662
C	2.48281	-3.72665	-0.94203
H	2.61187	-4.77889	-1.16546
C	3.56248	-2.85937	-1.08839
C	3.41750	-1.49113	-0.85632
H	4.25305	-0.82180	-1.02992
C	2.18357	-0.95556	-0.45914
C	1.16899	-1.89537	-0.24660
C	-0.16969	-4.39178	1.17771
H	-1.09094	-4.95350	1.35579
H	0.68327	-4.98833	1.51327
H	-0.20548	-3.47920	1.77520
C	0.01097	-5.35674	-1.11435
H	0.11020	-5.16366	-2.18446
H	0.85159	-5.97526	-0.79284
H	-0.89964	-5.93604	-0.94778
C	-2.81734	1.35254	-0.47148
C	-3.76247	1.32106	0.57378
C	-4.82639	2.21798	0.55020
H	-5.55707	2.19198	1.35281
C	-4.95910	3.14688	-0.46505
H	-5.79181	3.84306	-0.46660
C	-4.01037	3.19316	-1.47392
H	-4.10911	3.93494	-2.25759
C	-2.93699	2.30863	-1.49917
C	-1.92101	2.35037	-2.61887
H	-0.94963	2.17930	-2.14728
C	-1.85526	3.69822	-3.32342
H	-2.77059	3.91153	-3.88517
H	-1.69130	4.51151	-2.61225
H	-1.02982	3.70388	-4.03799
C	-2.15726	1.23172	-3.63199
H	-1.37828	1.24351	-4.40140
H	-2.14588	0.25218	-3.15386
H	-3.12556	1.35816	-4.12818
C	-3.65141	0.34801	1.72877
H	-2.71067	-0.19096	1.61310
C	-3.59270	1.07257	3.07055
H	-4.52884	1.59896	3.28301
H	-3.41963	0.35534	3.87795
H	-2.77582	1.79418	3.09079
C	-4.78935	-0.67151	1.72110
H	-4.81087	-1.23249	0.78531
H	-4.66476	-1.38429	2.54204
H	-5.75819	-0.17766	1.85026
C	2.86383	1.30366	-0.45607

C	3.01354	1.99384	-1.67315
C	4.05857	2.90106	-1.80907
H	4.18195	3.43883	-2.74261
C	4.94640	3.13224	-0.77019
H	5.75860	3.84142	-0.89479
C	4.78145	2.46447	0.43010
H	5.46600	2.65783	1.25034
C	3.74397	1.55404	0.61160
C	3.58180	0.85987	1.94560
H	2.64394	0.30418	1.90660
C	4.71233	-0.13365	2.20724
H	5.68016	0.37705	2.25373
H	4.55519	-0.64395	3.16242
H	4.76120	-0.89057	1.42228
C	3.46854	1.86113	3.09114
H	2.67786	2.58648	2.89718
H	3.21931	1.34303	4.02126
H	4.40728	2.40332	3.24559
C	2.06916	1.71858	-2.82069
H	1.12381	1.40551	-2.37161
C	2.57045	0.56254	-3.68555
H	3.53018	0.81714	-4.14804
H	2.70824	-0.34404	-3.09493
H	1.85432	0.34419	-4.48407
C	1.79161	2.94578	-3.67684
H	1.02604	2.71177	-4.42125
H	1.43363	3.77882	-3.06808
H	2.68115	3.27417	-4.22345
C	-0.08898	-0.19310	3.08122
H	-0.05814	-1.18085	2.62575
C	-0.06739	0.93496	2.25484
C	-0.10254	2.17535	2.90317
H	-0.08623	3.08275	2.30372
C	-0.15272	2.27983	4.28791
H	-0.17197	3.25736	4.76197
C	-0.17550	1.13479	5.07105
H	-0.21413	1.21455	6.15450
C	-0.14794	-0.12221	4.47176
H	0.09059	2.46437	-0.23673
C	-4.69639	-3.20410	-2.24331
H	-5.01285	-2.55278	-3.06249
H	-4.58977	-4.21750	-2.63676
H	-5.50805	-3.21638	-1.50791
C	4.90578	-3.39257	-1.49966
H	5.59395	-3.42993	-0.64844
H	4.82304	-4.40406	-1.90374
H	5.36799	-2.75845	-2.26103
C	-0.21445	-1.37221	5.30096
H	0.26738	-1.23631	6.27293
H	-1.25306	-1.66665	5.48899
H	0.27253	-2.20765	4.79242

Toluene activation TS, para

110

C	-2.52520	-0.95077	-2.67152
C	-2.23962	0.02694	-1.59954
C	-3.22695	1.12288	-1.61194
C	-4.51504	0.84689	-1.99149
C	-4.89128	-0.37373	-2.59222
C	-3.83243	-1.18261	-3.03443
Al	-0.58457	-0.01094	-0.67299
N	-0.06640	1.52263	0.32216
C	-0.96423	2.50066	0.81528
C	-1.85439	2.18591	1.85798
C	-2.72257	3.16916	2.32408
C	-2.73538	4.43934	1.78091
C	-1.89221	4.72709	0.72109
C	-1.02061	3.77522	0.20725
C	-1.94201	0.80479	2.46560
C	-3.30254	0.16805	2.18565
C	-0.23829	4.12441	-1.04477
C	-1.17747	4.32498	-2.23388
O	1.32498	0.16111	-1.39803
C	1.99515	-1.06831	-1.39574
C	3.34330	-1.09191	-1.65712
C	4.01427	0.26288	-1.88425
C	3.39451	1.21175	-0.85274
C	2.03719	1.11920	-0.66841
C	3.98498	-2.32429	-1.52373
C	3.27648	-3.43788	-1.06869
C	1.94887	-3.31806	-0.66554
C	1.28108	-2.09115	-0.76609
C	1.27157	1.83320	0.25484
C	1.98333	2.76283	1.02101
C	3.35709	2.92465	0.85614
C	4.06382	2.14903	-0.06507
N	0.07838	-1.73846	-0.21406
C	-0.34977	-2.43704	0.94667
C	-1.55417	-3.16348	0.92312
C	-1.99949	-3.75619	2.10091
C	-1.28021	-3.64557	3.28002
C	-0.08845	-2.94365	3.28917
C	0.39851	-2.33075	2.13709
C	3.96008	-4.77081	-0.97012
C	3.68381	0.77748	-3.29563
C	5.52428	0.17467	-1.72786
C	4.07716	3.95841	1.67402
C	-2.32159	-3.33629	-0.36346
C	-1.78604	-4.53521	-1.14535
C	1.70726	-1.56807	2.22107
C	2.85838	-2.48744	2.62435
C	1.62427	-0.36776	3.15828
C	-3.82716	-3.43815	-0.17394
C	0.65529	5.34605	-0.84504
C	-1.65458	0.81793	3.96510
H	-2.40995	-0.55378	-0.48119
H	1.44480	-4.15351	-0.19221
H	5.04377	-2.41739	-1.73280

H	5.13633	2.27450	-0.15323
H	1.44919	3.35001	1.76002
H	4.11943	1.76899	-3.44418
H	2.60672	0.84863	-3.44922
H	4.09279	0.09630	-4.04641
H	5.80485	-0.18350	-0.73531
H	5.97872	1.15514	-1.88577
H	5.94406	-0.50393	-2.47356
H	-2.93352	-4.30610	2.09248
H	-1.65003	-4.10594	4.19063
H	0.47389	-2.85319	4.21341
H	1.94115	-1.17585	1.23297
H	0.87185	0.34138	2.81521
H	2.58491	0.15459	3.18444
H	1.37024	-0.66974	4.17849
H	3.80605	-1.94191	2.59570
H	2.93586	-3.33787	1.94492
H	2.72130	-2.87154	3.63976
H	-2.14971	-2.45861	-0.98946
H	-4.12891	-4.38968	0.27885
H	-4.31591	-3.35079	-1.14656
H	-4.19948	-2.61921	0.44517
H	-0.72116	-4.42092	-1.36082
H	-2.31414	-4.62188	-2.09918
H	-1.92525	-5.46682	-0.58564
H	-3.40945	2.92275	3.12704
H	-3.41721	5.19302	2.16042
H	-1.93577	5.70638	0.25508
H	0.40816	3.28397	-1.29232
H	0.06229	6.24266	-0.64142
H	1.24519	5.53621	-1.74642
H	1.34613	5.19816	-0.01263
H	-1.77982	3.43309	-2.41994
H	-0.59862	4.54260	-3.13700
H	-1.85942	5.16322	-2.06227
H	-1.18042	0.17887	1.99334
H	-0.70065	1.30068	4.18806
H	-1.61870	-0.20562	4.34792
H	-2.43743	1.35559	4.50861
H	-4.09553	0.69865	2.72276
H	-3.30211	-0.87414	2.51559
H	-3.54163	0.19024	1.11999
H	-2.97863	2.10454	-1.22246
H	-5.26854	1.63029	-1.89488
H	-4.04775	-1.96765	-3.76293
H	-1.72078	-1.48044	-3.17486
H	3.69657	3.98981	2.69766
H	5.14962	3.75636	1.71460
H	3.94412	4.95807	1.24666
H	5.04394	-4.65634	-0.89750
H	3.61480	-5.32908	-0.09651
H	3.75031	-5.38451	-1.85219
C	-6.31625	-0.67526	-2.94045
H	-6.86038	-1.21405	-2.14948
H	-6.88580	0.24244	-3.13760
H	-6.38107	-1.29915	-3.84118

Toluene activation product, para

110

C	-0.07890	-1.78534	-2.51192
C	-0.08422	-2.10032	-1.14819
C	-0.15111	-3.46502	-0.84453
C	-0.21347	-4.44817	-1.82504
C	-0.21375	-4.10946	-3.17426
C	-0.14006	-2.75773	-3.50212
Al	0.01203	-0.79369	0.37068
N	-1.75195	0.06892	0.61542
C	-2.82210	-0.64060	1.21064
C	-2.94223	-0.70437	2.61282
C	-4.02217	-1.38491	3.16573
C	-4.97681	-1.99296	2.36639
C	-4.84330	-1.94290	0.99130
C	-3.77288	-1.28443	0.39320
C	-1.91944	-0.02277	3.49422
C	-1.85739	-0.59945	4.90175
C	-3.66078	-1.29111	-1.11688
C	-3.62944	-2.71240	-1.67197
O	-0.00252	0.97764	-0.98794
C	1.18675	1.66772	-1.04596
C	1.27213	2.87151	-1.69697
C	0.00549	3.38949	-2.37538
C	-1.18393	2.98955	-1.50354
C	-1.11826	1.77407	-0.87107
C	2.51913	3.50748	-1.68160
C	3.59272	2.92426	-1.01344
C	3.43537	1.72598	-0.31615
C	2.19470	1.07283	-0.27905
C	-2.08119	1.23682	-0.01093
C	-3.24738	2.00193	0.12900
C	-3.38125	3.23059	-0.51828
C	-2.35537	3.73181	-1.31554
N	1.82871	-0.02099	0.44602
C	2.85626	-0.66831	1.16933
C	3.72707	-1.55660	0.51375
C	4.75713	-2.14903	1.23908
C	4.92405	-1.89008	2.58776
C	4.04569	-1.03429	3.23356
C	3.00827	-0.41506	2.54514
C	4.94255	3.58400	-1.03780
C	-0.13897	2.69092	-3.73941
C	0.06150	4.89542	-2.59177
C	-4.65652	4.00825	-0.35422
C	3.56276	-1.88004	-0.95452
C	4.70373	-1.30444	-1.79131
C	2.07474	0.54498	3.24606
C	2.59396	1.98023	3.16724
C	1.79018	0.15816	4.69014
C	3.42586	-3.38146	-1.18841

C	-4.78321	-0.48708	-1.77116
C	-2.14274	1.48744	3.54803
H	0.07166	-1.67215	1.74186
H	4.26625	1.31478	0.24675
H	2.65777	4.45795	-2.18277
H	-2.47425	4.69970	-1.78777
H	-4.04195	1.63921	0.77219
H	-1.05699	3.01780	-4.23575
H	-0.18445	1.60750	-3.61642
H	0.71661	2.92816	-4.37792
H	0.16443	5.42932	-1.64487
H	-0.84511	5.24340	-3.09128
H	0.90556	5.15807	-3.23297
H	5.43423	-2.83240	0.73576
H	5.73033	-2.36230	3.14009
H	4.17047	-0.84658	4.29427
H	1.12989	0.50827	2.69896
H	1.41903	-0.86666	4.75841
H	1.03256	0.82554	5.10916
H	2.68004	0.24646	5.32122
H	1.88529	2.66915	3.63770
H	2.73754	2.29400	2.13254
H	3.55386	2.07047	3.68711
H	2.63259	-1.41492	-1.28339
H	4.35569	-3.91123	-0.95624
H	3.17531	-3.57846	-2.23444
H	2.62728	-3.79949	-0.57486
H	4.76866	-0.22089	-1.67603
H	4.54505	-1.52470	-2.85137
H	5.66462	-1.74009	-1.49711
H	-4.12137	-1.44301	4.24318
H	-5.81472	-2.51467	2.81785
H	-5.57851	-2.43539	0.36229
H	-2.71041	-0.82334	-1.37591
H	-5.76068	-0.92832	-1.54940
H	-4.65851	-0.47787	-2.85827
H	-4.78468	0.54645	-1.42102
H	-2.82862	-3.29357	-1.21452
H	-3.44753	-2.69023	-2.75014
H	-4.57848	-3.23060	-1.50044
H	-0.95030	-0.20445	3.02205
H	-2.12921	1.92835	2.55120
H	-1.35886	1.96767	4.14288
H	-3.10848	1.71868	4.01030
H	-2.77055	-0.39245	5.46935
H	-1.02777	-0.15017	5.45109
H	-1.70279	-1.68102	4.88236
H	-0.15152	-3.76857	0.20028
H	-0.26176	-5.49673	-1.53939
H	-0.12993	-2.46488	-4.54991
H	-0.02083	-0.74364	-2.81694
H	-4.97229	4.03680	0.69217
H	-4.53999	5.03711	-0.70212
H	-5.47285	3.55404	-0.92607
H	4.86979	4.62135	-1.37228
H	5.40523	3.57727	-0.04719
H	5.62529	3.06277	-1.71732
C	-0.32157	-5.16078	-4.23985
H	-1.36729	-5.34603	-4.50988
H	0.20126	-4.85919	-5.15120
H	0.10147	-6.11099	-3.90438

References

S1. J. Hicks, P. Vasko, J. M. Goicoechea, S. Aldridge, *Nature* **2018**, *557*, 92-95.

S2. J. Cosier, A. M. Glazer, *J. Appl. Cryst.* **1986**, *19*, 105-107.

S3. CrysAlisPro, Agilent Technologies, Version 1.171.35.8.

S4. G. M. Sheldrick, SHELX-2018 package (**2018**).

S5. L. J. Barbour, *J. Supramol. Chem.* **2001**, *1*, 189-191.

S6. Gaussian 16, Revision C.01, M. J. Frisch, G. W. Trucks, H. B. Schlegel, G. E. Scuseria, M. A. Robb, J. R. Cheeseman, G. Scalmani, V. Barone, G. A. Petersson, H. Nakatsuji, X. Li, M. Caricato, A. V. Marenich, J. Bloino, B. G. Janesko, R. Gomperts, B. Mennucci, H. P. Hratchian, J. V. Ortiz, A. F. Izmaylov, J. L. Sonnenberg, D. Williams-Young, F. Ding, F. Lipparini, F. Egidi, J. Goings, B. Peng, A. Petrone, T. Henderson, D. Ranasinghe, V. G. Zakrzewski, J. Gao, N. Rega, G. Zheng, W. Liang, M. Hada, M. Ehara, K. Toyota, R. Fukuda, J. Hasegawa, M. Ishida, T. Nakajima, Y. Honda, O. Kitao, H. Nakai, T. Vreven, K. Throssell, J. A. Montgomery, Jr., J. E. Peralta, F. Ogliaro, M. J. Bearpark, J. J. Heyd, E. N. Brothers, K. N. Kudin, V. N. Staroverov, T. A. Keith, R. Kobayashi, J. Normand, K. Raghavachari, A. P. Rendell, J. C. Burant, S. S. Iyengar, J. Tomasi, M. Cossi, J. M. Millam, M. Klene, C. Adamo, R. Cammi, J. W. Ochterski, R. L. Martin, K. Morokuma, O. Farkas, J. B. Foresman, and D. J. Fox, Gaussian, Inc., Wallingford CT, 2019.

S7. (a) J. P. Perdew, K. Burke, M. Ernzerhof, *Phys. Rev. Lett.*, **1996**, *77*, 3865-3868; (b) J. P. Perdew, K. Burke, M. Ernzerhof, *Phys. Rev. Lett.*, **1997**, *78*, 1396; (c) C. Adamo, V. Barone, *J. Chem. Phys.*, **1999**, *110*, 6158-6169.

S8. (a) A. Schäfer, H. Horn, R. Ahlrichs, *J. Chem. Phys.* **1992**, *97*, 2571-2577; (b) A. Schäfer, H. Horn, R. Ahlrichs, *J. Chem. Phys.* **1994**, *100*, 5829-5835.

S9. S. Grimme, S. Ehrlich, L. Goerigk, *J. Comp. Chem*. **2011**, *32*, 1456-1465.

S10. NBO 7.0. E. D. Glendening, J. K. Badenhoop, A. E. Reed, J. E. Carpenter, J. A. Bohmann, C. M. Morales, P. Karafiloglou, C. R. Landis, and F. Weinhold, Theoretical Chemistry Institute, University of Wisconsin, Madison, WI, 2018.
